# Galaxy and MEAN Stack to Create a User-Friendly Workflow for the Rational Optimization of Cancer Chemotherapy

**DOI:** 10.3389/fgene.2021.624259

**Published:** 2021-02-18

**Authors:** Jorge Guerra Pires, Gilberto Ferreira da Silva, Thomas Weyssow, Alessandra Jordano Conforte, Dante Pagnoncelli, Fabricio Alves Barbosa da Silva, Nicolas Carels

**Affiliations:** ^1^Plataforma de Modelagem de Sistemas Biológicos, Center for Technology Development in Health (CDTS), Oswaldo Cruz Foundation (FIOCRUZ), Rio de Janeiro, Brazil; ^2^Informatic Department, Free University of Brussels (ULB), Brussels, Belgium; ^3^Laboratório de Modelagem Computacional de Sistemas Biológicos, Scientific Computing Program, FIOCRUZ, Rio de Janeiro, Brazil; ^4^Instituto COI, Rio de Janeiro, Brazil

**Keywords:** systems biology, translational oncology, personalized medicine, Galaxy, MEAN stack, angular, protein–protein network, Shannon entropy

## Abstract

One aspect of personalized medicine is aiming at identifying specific targets for therapy considering the gene expression profile of each patient individually. The real-world implementation of this approach is better achieved by user-friendly bioinformatics systems for healthcare professionals. In this report, we present an online platform that endows users with an interface designed using MEAN stack supported by a Galaxy pipeline. This pipeline targets connection *hubs* in the subnetworks formed by the interactions between the proteins of genes that are up-regulated in tumors. This strategy has been proved to be suitable for the inhibition of tumor growth and metastasis *in vitro*. Therefore, Perl and Python scripts were enclosed in Galaxy for translating RNA-seq data into protein targets suitable for the chemotherapy of solid tumors. Consequently, we validated the process of target diagnosis by (i) reference to subnetwork entropy, (ii) the critical value of density probability of differential gene expression, and (iii) the inhibition of the most relevant targets according to TCGA and GDC data. Finally, the most relevant targets identified by the pipeline are stored in MongoDB and can be accessed through the aforementioned internet portal designed to be compatible with mobile or small devices through Angular libraries.

## Introduction

The worldwide estimate of people diagnosed with cancer was 18.1 million in 2017^1^ and it is predicted by the *World Health Organization* (WHO) to be 27 million new cases worldwide by 2030. On its own, breast cancer (BC) continues to be among the most frequent cancer around the world alongside the prostate one. Moreover, BC, alone accounts for almost 2.1 million new cases diagnosed annually worldwide, causing an estimate of 600,000 deaths every year (Bray et al., 2018). Because of these dire statistics, BC has received huge attention from both the academic and the industry, which resulted in a large corpus of publication (culminating at 25,000 in 2019^2^) and publicly available datasets.

In addition, the well-known heterogeneity of breast cancer has justified the genomic study of tumors on a large scale in search for tumor subtypes that could allow a better understanding of the tumor biology and could serve as support for the establishment of genetic signatures, which, when validated in clinical trials, could pave the way for an increasingly specific and more precise treatment than the clinical parameters currently in use.

It is a more in-depth knowledge of tumor biology that has allowed for greater individualization of available treatments and has made it possible to overcome the relapse and resistance eventually observed with traditional treatments (Naito and Urasaki, 2018). In addition, clinical experience has shown that knowledge of the individual characteristics of each tumor may contribute to better therapeutic results with less toxicity.

According to the *one-size-fits-all* approach of chemotherapy, treatment should fit every individual of a population. As a consequence, it is intrinsically imprecise since it does not take into account the genetic peculiarities of each patient. Thus, a one-size-fits-all treatment approach does not work for everyone and may cause harmful side effects. By contrast, *personalized oncology*, which can be placed into a wider paradigm shift called *personalized medicine*, involves the tailoring of medical treatment to the individual characteristics or symptoms and responses of a patient during all stages of care.

The paradigm of one-size-fits-all treatment is now undergoing a shift toward personalized oncology with the identification of molecular pathways predicting both tumor biology as well as response to therapy. Most of those achievements have been inserted into mathematical and computational models by different groups, which can be used to test therapies and hypothesis; the one presented herein fall into this category.

A *new taxonomy* of disease based on molecular and environmental determinants rather than signs and symptoms has been proposed (Collins and Varmus, 2015). The paradigm revolution lies in the change from a clinician selecting a generic therapy on a heuristic basis to one based on molecular facts, a process called *evidence-based medicine* (Masic et al., 2008).

The tools of systems biology made it possible to analyze the huge amount of data delivered by high throughput technologies (broadly named Big Data, Willems et al., 2019). At the moment, the most common strategy for implementing high throughput technologies in oncology is to map mutations that promote suppressor and oncogenes (Guo et al., 2014; Campbell et al., 2020), which is a typical activity of *pharmacogenomics*. Briefly, pharmacogenomics aims at understanding why individuals respond differently to medicines on a genetic level. Consequently, it enables one to predict an individual’s response to a drug according to genetic information and allows one to choose the most appropriate medication according to an individual’s genetic composition. Furthermore, when the molecular diagnosis is performed, targeted therapy is designed for acting on specific molecular targets supposed to be relevant for the tumor under consideration (Wilsdon et al., 2018). Notwithstanding all the knowledge we have gathered so far, the relevance of a drug target is not obvious, and many criteria were pursuit in that quest (Catharina et al., 2018).

The development of personalized medicine is directly related to the availability of high-throughput technologies. High-throughput techniques, such as microarray, *RNA sequencing* (RNA-seq), and nanoString^3^ are important tools for the characterization of tumors and their adjacent non-malignant tissues (Finak et al., 2006). Therefore, these techniques allow a better understanding of tumor biology (Carels et al., 2020). In particular, RNA-seq analysis through in silico methodologies demonstrated that each tumor is unique considering the protein profile of their up-regulated genes (Carels et al., 2015a).

Following the current state of the art, there are mainly two types of omics tests: (i) prognostic tests, which predicts a clinical outcome, and (ii) therapy guiding tests (theranostics), which enable the identification of patient subgroups with a similar response to a particular therapy (McShane and Polley, 2013). In this report, we focus on theranostics.

A variety of multigene assays are in clinical use or under investigation, which further defines the molecular characteristics of the cancers’ dominant biologic pathways. Even if there has been a growing use of biomarkers in clinical trials, the use of single-marker and panel tests is still limited (Vuckovic et al., 2016). Gaining insight into the molecular composition of each tumor is recommended for eliminating the misuse of ineffective and potentially harmful drugs.

Mapping gene alterations by reference to the genome is generally performed to characterize indirect relationships between tumor development and indels, mutations, hyper- or hypo-methylation. By contrast, the description of transcriptome, proteome, or metabolome allows the characterization of a molecular phenotype. Interestingly, most *companion diagnostics* (CD) for cancer characterization on the market are based on mutation profiling. Accordingly, CDs are expected to guide the application of a specific therapy supposed to be efficacious for a given patient’s condition (Verma, 2012). As a result, CDs allow the selection of a treatment that is more likely to be effective for each individual based on the genetic signatures of their tumors. Moreover, CDs are also developed for better predicting the patient response to a given treatment.

An approach based on molecular phenotyping recently proposed was the identification of the most relevant protein targets for specific therapeutic intervention in malignant BC cell lines (Carels et al., 2015a) based on the diagnosis of up-regulated interactome hubs. This strategy combined *protein-protein interactions* (PPI) and RNA-seq data for inferring (i) the topology of the signaling network of up-regulated genes in malignant cell lines and (ii) the most relevant protein targets therein. Hence, it has the benefit to allow the association of a drug to the entropy of a target and, additionally, to rank drugs according to their respective entropy by reference to their targets (Carels et al., 2015b).

Three concepts were considered in the approach followed by Carels et al. (2015a): (i) A vertex with a high expression level is more influential than a vertex with a low expression level. (ii) A vertex with a high connectivity level (hub) is more influential than a vertex with a low connectivity level. (iii) A protein target must be expressed at a significantly higher level in tumor cells than in the cells used as a non-malignant reference to reduce harmful side effects to the patient after its inhibition. It is worth mentioning that each combination of targets that most closely satisfied these conditions was found to be specific for its respective malignant cell lines. These statements were validated *in vitro* on a BC model by Tilli et al. (2016). These authors showed that the inactivation, by *small interfering RNA* (siRNA), of the five top-ranked hubs of connection (top-5) identified for MDA-MB-231, a triple-negative cell line of invasive BC, resulted in a significant reduction of cell proliferation, colony formation, cell growth, cell migration, and cell invasion. Inhibition of these targets in other cell lines, such as MCF-7 (non-invasive malignant breast cell line) and MCF-10A (non-tumoral cell line used as a control), showed little or no effect, respectively. In addition, the effect of joint target inhibition was greater than the one expected from the sum of individual target inhibitions, which is in line with the buffer effect of regulatory pathway redundancy in malignant cells (Tilli et al., 2016).

The signaling network of a biological system is scale-free (Albert et al., 2000), which means that few proteins have high connectivity values and many proteins have low connectivity values. As proven mathematically, the inhibition of proteins with high connectivity values has a greater potential for signaling network disruption than randomly selected proteins (Albert et al., 2000). This evidence was proven in silico by Conforte et al. (2019) in the particular case of tumor signaling networks.

In terms of systems biology, the inhibitory activity of a drug may be modeled by the removal of its corresponding protein target from the signaling network to which it belongs (Carels et al., 2015b; Conforte et al., 2019). The impact of vertex removal from a network can be evaluated by the use of the Shannon entropy, which has been proposed as a network complexity measure and applied by many authors to determine a relationship between network entropy and tumor aggressiveness. Breitkreutz et al. (2012), for instance, inferred a negative correlation between the entropy of networks made of genes documented in the *Kyoto Encyclopedia of Genes and Genomes* (KEGG^4^) database considering cancer types and their respective 5-year survival. The existence of this negative correlation was demonstrated later on by Conforte et al. (2019) using RNA-seq data from bench experiments stored in *The Cancer Genome Atlas* (TCGA now hosted by the *Genomic Data Commons Data Portal* – GDC Data Portal^5^).

The Shannon entropy (*H*) is given by formula 1

(1) $$H=-\sum_{k=1}^{n}p\left(k\right)log_{2}\left(p\left(k\right)\right)$$

where *p*(*k*) is the probability that a vertex with a connectivity value *k* occurs in the analyzed network.

The process of multistep mining of high throughput data can be cumbersome to handle by humans and needs translation into machine language and automation (Deelman et al., 2009). Thus, according to the scientific challenge, we developed codes in Perl and Python. To deal with assembling a workflow based on *heterogeneous programming*, i.e., a workflow including more than one programming language, we chose Galaxy (Afgan et al., 2018) that fit this purpose.

Since we believe that a molecular phenotyping strategy is worthwhile for complementing the genotyping approach, we described in this report how to perform the translation from RNA-seq data into therapy targets based on the process described in more detail in Conforte et al. (2019). The most relevant targets stored in MongoDB can be accessed through an internet portal written in JavaScript using the software bundle called MEAN stack and portable to mobile and small devices through Angular Flex-Layout library and *Lazy loading^6^* strategies as described by Fain and Moiseev (2018) and Holmes and Herber (2019).

## Materials and Methods

### Galaxy Pipeline

#### TCGA Data

The gene expression data were obtained as RNA-seq files from paired samples (control and tumor samples from the same patient) and downloaded from TCGA^7^ in February 2016 and from the GDC Data Portal^8^ in March 2020. The data selection followed two criteria: (i) for each cancer type, approximately 30 patients with paired samples were required to satisfy statistical significance; and (ii) the tumor samples had to be from a solid tumor. The data from TCGA and GDC are given in Table 1.

**TABLE 1:** RSEM-UQ from paired tumor-stroma data retrieved from TCGA and FPKM-UQ from GDC.

Tumor type	Abbreviation	OS^1^	TCGA, *n*^2^	GDC, *n*
Stomach adenocarcinoma	STAD	38	32	27
Lung adenocarcinoma	LUAD	40	57	57
Lung squamous cell carcinoma	LUSC	47	50	48
Liver hepatocellular carcinoma	LIHC	49	49	50
Kidney renal clear cell carcinoma	KIRC	63	71	71
Kidney renal papillary cell carcinoma	KIRP	75	32	31
Breast cancer	BRCA	82	72	46
Thyroid cancer	THCA	93	57	56
Prostate cancer	PRAD	98	51	50

*^1^OS: 5-years overall survival taken from Liu et al. (2018) according to Conforte et al. (2019), %. ^2^n: Sample size, number.*

In TCGA, gene expression values were given for 20,532 genes referred to as GeneSymbol, calculated by *RNA-seq through expectation maximization* (RSEM) (Mortazavi et al., 2008; Li and Dewey, 2011). Since they were normalized according to the upper quartile methods (formula 2) as reported in GDC documentation^9^, we denoted them as RSEM-UQ. In the case of GDC, gene expression values were given for 60,483 sequences, calculated by FPKM and referred to as Ensembl accession number. As those values were also normalized by upper quartile, they were denoted, here, as FPKM-UQ. We considered RNA-seq from BRCA and LUAD as non-significant because of inconsistencies between *raw counts* file names, which led to a final sample of 16 and 17 for LUAD and BRCA, respectively. The 14,126 genes for which the equivalence between GeneSymbols and UniProtKB could be obtained went through further analysis.

(2) $$N_{norm}=\frac{RC_{g}*10^{9}}{RC_{g75}*L}$$

where:

*RC*_*g*_: Number of reads mapped to the gene;

*RC_*g*75_*: The 75th percentile read count value for genes in the sample;

*L*: Length of the coding sequence in base pairs.

#### ArrayEXPRESS Data

Fastq files from RNA-seq of tumor-stroma paired samples from 14 PRAD^10^, and 18 *non-small cell lung cancer* (NSCLC)^11^, were retrieved from ArrayEXPRESS^12^. These files were compared to the proteins of the EBI’s interactome (see below) using BLASTx and processed through our pipeline to measure the average entropies of malignant up-regulated genes from both PRAD and NSCLC. The statistical significance of average entropy differences between PRAD and NSCLC was assessed through the Student’s *t*-test using formula 3:

(3) $$u_{obs}=\frac{\left|{\bar{x}}_{1}-{\bar{x}}_{2}\right|}{\sqrt{\frac{SCE_{1}}{n_{1}\left(n_{1}-1\right)}+\frac{SCE_{2}}{n_{2}\left(n_{2}-1\right)}}}$$

where:

${\bar{x}}_{i}$: The average of sample *i*;

*SCE*_*i*_: the sum of squared differences of sample *i*;

*n*_*i*_: the size of sample *i*.

Because sample sizes of PRAD (*n* = 14) and NSCLC (*n* = 18) were less than *n* = 20, *u*_*obs*_ was compared to the theoretical value *t_1–α/2_* of the Student’s distribution using the *k* degree of freedom calculated according to formula 4 (Welch, 1949; Dagnelie, 1970):

(4) $$k=\frac{{\left[\frac{SCE_{1}}{n_{1}\left(n_{1}-1\right)}+\frac{SCE_{2}}{n_{2}\left(n_{2}-1\right)}\right]}^{2}}{\frac{1}{n_{1}-1}{\left[\frac{SCE_{1}}{n_{1}\left(n_{1}-1\right)}\right]}^{2}+\frac{1}{n_{2}-1}{\left[\frac{SCE_{2}}{n_{2}\left(n_{2}-1\right)}\right]}^{2}}$$

with *n*_1_-1 < *k* < *n_1_* + *n*_2_-2.

### Identification of Hubs Among Genes Up-Regulated in Tumor Samples

To identify genes that were significantly differentially expressed in the tumor samples of patients, we subtracted gene expression values of control samples from their respective tumor paired samples. The resulting values were called differential gene expression. Negative differential gene expression values indicated higher gene expressions in control samples, while positive differential gene expression values indicated higher gene expressions in tumor samples.

The histogram of differential expression was normalized with the Python packages *scipy.* We used the probability density and cumulative distribution functions, respectively abbreviated as PDF and CDF, in the interval of differential gene expression from −20.000 to +20.000, to calculate the critical value corresponding to the one-tail cumulated probability *p* = 0.975, which corresponded to a *p*-value α = 0.025. We considered the genes as up-regulated when their differential expression was larger than the critical value corresponding to *p* = 0.975. The −20.000 to +20.000 range worked fine for the *p*-value and normalization conditions presented in this report. However, some normalization procedures flatten the probability distribution with Bayesian functions for variance minimization. Under these conditions, a *p*–value of 0.001 may represent a very large critical value of 80,000 or more, which would induce the scipy package to return “out of range.” To beat this challenge, we introduced the possibility of tuning the −20.000 to +20.000 range to allow the user to try other normalization conditions together with more restrictive *p*-values. However, for coherence, all the data produced in this report were obtained with critical values in the −20.000 to +20.000 range.

In a subsequent step, the protein–protein interaction (PPI) subnetworks were inferred for the proteins identified as products of up-regulated genes. The subnetworks were obtained by comparing these gene lists with the human interactome.

The human interactome (151,631 interactions among 15,526 human proteins with UniProtKB accessions) was obtained from the intact-micluster.txt file (version updated December 2017) accessed on January 11, 2018^13^.

We used the PPI subnetworks of up-regulated genes from each patient to identify each vertex (protein) degree through automated counting of their edges. These values were used to calculate the Shannon entropy of each PPI subnetwork as explained in the section “Shannon Entropy” below.

### Shannon Entropy

The Shannon entropy was calculated with formula 1, where *p*(*k*) is the probability of occurrence of a vertex with a rank order *k* (*k* edges) in the subnetwork considered. The subnetworks were generated automatically from gene lists found to be up-regulated in each patient.

### Validation Process

**FIGURE 1:**
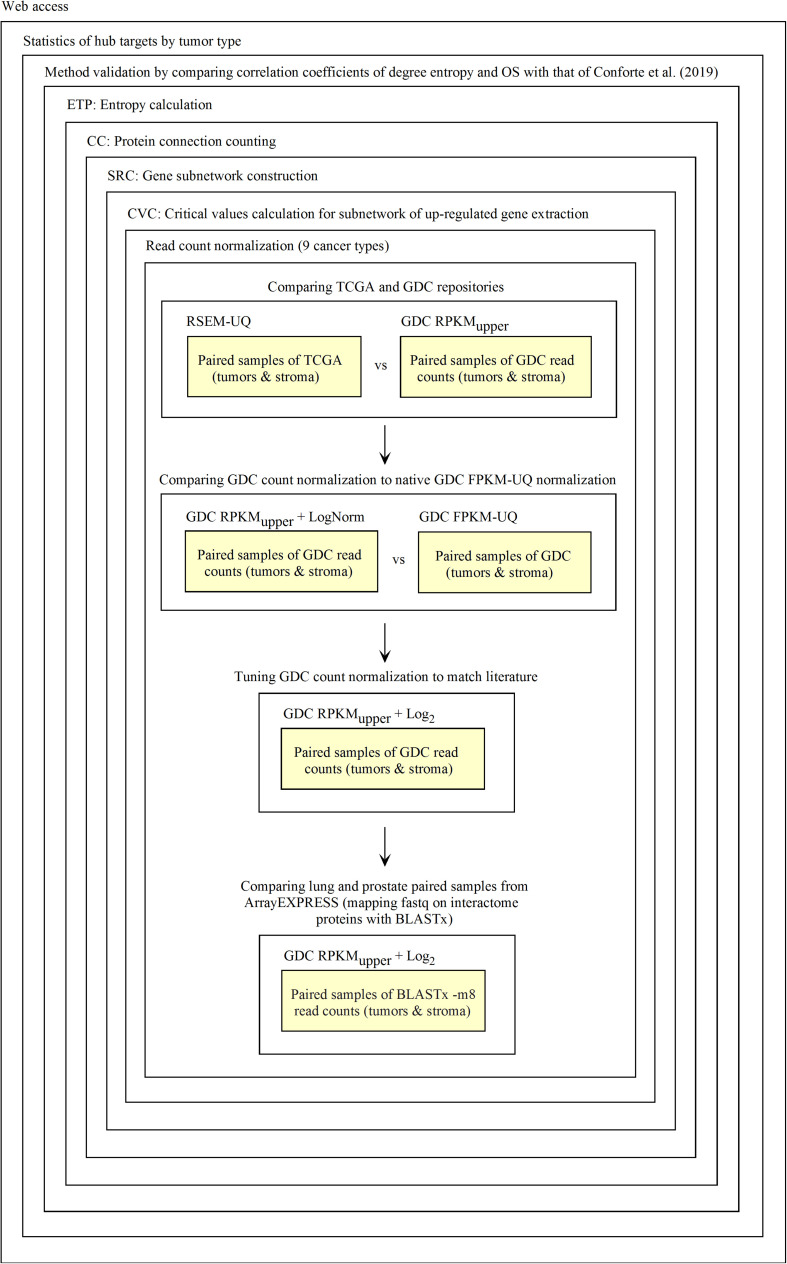
Process of Galaxy workflow validation.

The diagnosis of up-regulated genes with a higher vertex degree, which we considered as the most relevant target here, depends on how *fastq* and *raw count* files are processed. First, *fastq* reads need to be transformed into *raw counts* and, second, *raw counts* need to be normalized. For validating this process, we used the data of RSEM-UQ from TCGA as available in 2016 that we referenced to as TCGA RSEM-UQ below. When referring to the FPKM-UQ files from GDC accessed in March 2020, we denoted them as *GDC FPKM-UQ*. Since we had no access to the raw counts files of TCGA, we used the data from GDC. GDC provided the TCGA data in Bam format, *raw counts*, FPKM, and FPKM-UQ files. Since we knew the correlation between the entropy and the 5-years *overall survival* (OS) for nine cancer types as established from TCGA RSEM-UQ (Conforte et al., 2019), the validation challenge was (i) to normalize the GDC *raw counts* files (we characterized this step as RPKM_upper_, see the description below) from tumors of the nine cancer types; (ii) to compare the RPKM_upper_ normalization to the TCGA RSEM-UQ for critical value, number of up-regulated genes, and the correlation between entropy and 5-years OS as well as targets; (iii) to compare RPKM_upper_, TCGA RSEM-UQ and GDC FPKM-UQ for critical value, number of up-regulated genes, the correlation between entropy and 5-years OS, and targets, and (iv) to optimize RPKM_upper_ by log transformation for target selection given the maximization of the correlation coefficient of the relationship between entropy and 5-years OS. Having this process validated, it might be applied to any method of read counting from *fastq* file by read mapping. This process is summarized in Figure 1.

As TCGA, GDC uses the RSEM methodology to map reads to reference genes. Here, instead of using the human genome sequence GRCh38.d1.vd1^14^, we used the proteins sequences from UniprotKB as a reference. Since only about 80% of the proteins from the EBI’s interactome referenced by UniprotKB matched the *consensus coding sequences* (CCDS)^15^ of Ensembl, we decided to map reads in *fastq* files directly with the proteins sequences of the intact-micluster interactome using BLASTx. Thus, in the first instance, the exercise of validation concerned the processing of *raw counts* into RPKM-UQ output.

For *raw count* normalization, we used a modified version of the RPKM formula (5):

(5) $$RPKM=\frac{RC_{g}*10^{9}}{RC_{pc}*L}$$

where:

*RC*_*g*_: Number of reads mapped to the gene;

*RC*_*pc*_: Number of reads mapped to all protein-coding genes;

*L*: Length of the coding sequence in base pairs.

RPKM is relative to the total number of reads, which is a linear expectation. Quantile normalization (Bolstad et al., 2003) forces the distribution of the normalized data to be the same for each sample by replacing each quantile with the average quantile across all samples. Instead, one may focus on a specific quantile. For instance, the upper quartile normalization (Bullard et al., 2010) divides each read count by the 75th percentile of the read counts in its sample. However, the gene frequency (*y*) according to the gene expression (*x*) follows a power law (the relationship of log(y) and log(x) is linear, data not shown) (see also Balwierz et al., 2009; Awazu et al., 2018). RPKM, as defined in formula 5, does not take the non-linearity associated to large expression level into account. By contrast, the *upper quartile* normalization enables us to take the non-linearity associated with extreme expression values into account. Formula 5 can be written as formula 6:

(6) $$RPKM_{upper}=\frac{RC_{g}*10^{9}}{L*\left(RC_{pc}-\left(\delta *RC_{pc)}\right)\right)}$$

where δ is a tuning factor.

For δ = 0, formula 6 is equivalent to RPKM (formula 5) and for δ = 0.25, it is equivalent to a *upper quartile* normalization. In this work, we used δ = 0.05 because it optimized the coefficient of correlation between entropy and 5-years OS.

It appeared that in addition to the TCGA RSEM-UQ (accessed in 2016), GDC (accessed in March 2020) implemented a correction for false positive minimization (Anders and Huber, 2010; Love et al., 2014; Holmes and Huber, 2019). The result of this minimization is a flatten power law of gene expression with an effect similar to that of formula (7):

(7) $$LogNorm=C*x_{i}*\left(log_{b}\left(log_{b}\left(x_{i}+1\right)\right)+1\right)$$

where:

*C*: is a constant that was set to 20 to optimize the coefficient of correlation of the relationship between entropy and 5-years OS;

*x*_*i*_: is the RPKM_upper_ value of the *i*_*th*_ element;

*b*: is the base of the logarithm, which was set to 1.1.

As can be seen from formula 7, the FPKM-UQ output follows a *log-log* relationship except for the variance that is stabilized by a Bayesian process.

For assessing the efficiency of TCGA *raw counts* processing according to formula 6, we tabulated the sample size of subnetworks of up-regulated genes as well as the critical values obtained for PDF = 0.975. This process was performed by calculating RPKM_upper_ on the *raw counts* available from GDC, and compared the critical values to those obtained from GDC FPKM and TCGA RSEM-UQ. We also compared the correlation between entropy and 5-years OS obtained with *raw counts* normalized with RPKM_upper_ to that obtained by using the TCGA RSEM-UQ. Finally, we compared the most relevant targets obtained from both processes.

In the case of the GDC FPKM-UQ, one more step was necessary since the *raw counts* sequentially processed through formula 6 and 7 had to be compared to FPKM-UQ data available from the GDC portal. Again, we compared the performance of processing *raw counts* with formula 6 and 7 to GDC FPKM-UQ data considering (i) the critical values for PDF = 0.975, (ii) the subnetwork size of up-regulated genes, (iii) the correlation of entropy vs. 5-years OS, and (iv) the list of most relevant targets obtained through both processes.

Finally, we also compared the performance of sequentially processing *raw counts* through formula 6 and 8 (formula 8 is derived from Cloonan et al., 2008) by using the same measures as just described (i to iv). We applied this formula because we noticed that it optimized the coefficient of correlation of the relationship between entropy and 5-years OS.

(8) $$Log2=x_{i}\left(log_{b}\left(x_{i}+1\right)\right)$$

where:

*x*_*i*_: is the RPKM_upper_ value of the *i*_*th*_ element;

*b*: is the base of the logarithm, which was set to 2.

### Galaxy Scripts

Galaxy is a scientific open-source workflow platform that aims at helping users to perform repetitive and complex operations over large datasets. With Galaxy, users can visually create processing pipelines reproducing the data flow over programs and datasets that are viewed as interconnected box objects. Additionally, Galaxy is written in Python and JavaScript, but has an XML like interface able to transfer the processing flux to other languages. Galaxy comes with a rather large initial set of tools that can be added to the desktop according to simulation demands. Internally, every Galaxy tool is made up of a XML file that describes its functionalities and interface. Once XML interfaces are programmed, Galaxy is very simple to operate in an object-oriented mode by linking input data with scripts together.

By means of a specific script (see below), Galaxy can store data in MongoDB, which is a non-relational object-oriented database (NoSQL) (Bradshaw et al., 2019). MongoDB can be accessed through Angular, which serves as a frontend framework for users (the physician or/and technician operating the system) (Fain and Moiseev, 2018).

As outlined in the introduction of this report, our Galaxy workflows are derived from the agglomeration of Perl scripts (except for CVC.py) that were written for previous reports (Carels et al., 2015a; Conforte et al., 2019). These tools are as follow:

(1) *Count Connections* (CC) counts the number of connections that each protein has with their neighbors in a subnetwork of up-regulated genes. CC is an intermediate step to compute the entropy.
(2) *Critical Value Calculation* (CVC) computes a critical value according to the normal distribution that fits the observed data and a probability level informed by the user. All genes with expression values above the critical value, used here as a threshold, are considered as up-regulated.
(3) *Differentially Expressed Genes List* (DEGL) computes de differential gene expression between RNA-seq data from tumoral and control samples (tumor minus control).
(4) *Entropy Calculation* (ETP) computes the Shannon entropy corresponding to a subnetwork. Here, we typically considered the subnetworks of genes that are up-regulated in tumors.
(5) *Translation of Gene Symbol into UniProt KB accession numbers* (GS2UP). Former TCGA data files identified genes by gene Symbol, while the interactome from EBI (the intact-micluster.txt file) uses UniProtKB accession numbers. GS2UP translates the gene symbols to UniProtKB accession numbers to build the subnetwork of up-regulated genes.
(6) *Translation from Ensembl into UniProt KB accession numbers* (Ensembl2UP). GDC data files identify genes by reference to Ensembl, while the interactome from EBI (the intact-micluster.txt file) uses UniProtKB accession numbers. Ensembl2UP translates the Ensembl to UniProtKB accession number to build the subnetwork of up-regulated genes.
(7) *Protein To Total Connections Sorted* (PTTCS) sorts the file of malignant up-regulated genes according to the level of connectivity found for their respective protein in descending order.
(8) Subnetwork Construction (SRC) computes a subnetwork of proteins based on a gene list by reference to the intaractome; here, the gene list is typically the list of up-regulated genes.
(9) *Reads Per Kilobase Million – Upper Normalization* (RPKM_upper_) computes de normalization of RNA-seq data according to formula 6.
(10) *Double Logarithm Transformation* (LogNorm) computes de normalization of RPKM_upper_ data according to formula 7.
(11) *Base 2 Logarithm Transformation* (Log2) computes de normalization of RPKM_upper_ data according to formula 8.
(12) *PTTCS to MongoDB* (P2M) computes the data storage within MongoDB.

These tools can be downloaded from GitHub: https://github.com/BiologicalSystemModeling/Theranostics under the MIT License, however, the concept of theranostics based on this approach is under the regulation of intellectual property number BR1020150308191 for Brazil.

### Pipeline Scaling

To investigate how the pipeline scales, we processed the GDC raw counts data using an AMD Ryzen 9 3900X (4.6 GHz) CPU with 20 threads dedicated to Galaxy and 64 GB RAM. First, we chose LUSC and PRAD tumors as representing high entropy (low OS) and low entropy (high OS) cancer types, respectively. In these two cases, we could exactly compare their scaling until 45 patients by increments of five. For STAD, LIHC, THCA, and KIRC, we measured the processing time for only two patient numbers (15 and 25). We also analyzed the statistical significance of the difference in processing speed observed for entropy and PTTCS pipeline for 25 patients with the Student’s *t*-test. Considering the pipeline for hub diagnosis from BLASTx output, we only had access to a small number of patients, which limited the power of the experiment. We compared 3, 6, 9, 12 patients in PRAD and NSCLC from ArrayEXPRESS (see above).

### Web Application

As outlined in the introduction, we aimed at releasing a tool based on a phenotyping approach for the rational therapy of cancer. At the moment, the current approach of cancer therapy is still largely based on mutation mapping (genotyping approach), but the potential benefits of integrating RNA-seq data must be considered and this is the purpose of this report.

When producing a bioinformatic application, it is necessary to validate it according to some objective criterion. As presented in the previous section, we chose degree entropy as such a criterion for the validation of the Galaxy pipeline. Galaxy enabled us to test the performance of several configurations for optimizing the correlation between the degree entropy of up-regulated subnetworks and the patient’s 5-years overall survival.

However, a website is necessary to make this tool available to the medical community and its development makes part of another step of validation that is its acceptance by professionals. Below, we briefly describe the technologies that we used to build the web site and then described how we implemented them through forms for data submission.

### MEAN Stack

Both MongoDB and Angular are part of the MEAN stack (MEAN for M of MongoDB, E of Express.js, A of Angular, and N of Node.js). The use of MongoDB with Node.js, its native driver, is facilitated by the Mongoose^16^ library. Mongoose, amongst other benefits, allows (i) the use of JavaScript as a programming language, which save the need for database programming, (ii) the modeling of data before their saving into MongoDB, and (iii) the *horizontal scaling^17^*, which means that one can expand storage capacity without the need of multiple structural changes. This last feature decreases the cost of prototyping and expansion. It also enables one to work with several database connections simultaneously.

Node.js is part of the MEAN stack that we used to build the backend of the web application; it is the server used to connect the database and the frontend. Essentially, Node.js is a framework that is used to create servers and has its own HTTP handler (Holmes and Herber, 2019), which eliminates the need of other intermediate libraries.

The MEAN also included Express.js, a JavaScript-based library whose purpose is to facilitate the exploration of the Node.js functionalities (e.g., creating routes).

In addition to JavaScript, Angular also allows programming in TypeScript, which includes the concept of *variable type* and a set of internal libraries (e.g., RxJS for asynchronous programming). Furthermore, Angular offers compatibility with many web development libraries, such as Bootstrap, jQuery, and Forms.

MEAN stack elements have JavaScript as a common programming language and *JavaScript Object Notation* (JSON) as a common file exchange format. Except for Angular which is a frontend technology, MongoDB, Express.js, and Node.js run on the server-side, as so they are generally classified as the ‘backend’ of a web application (Holmes and Herber, 2019).

Our web application has been deployed in a cloud environment using Heroku^18, 19^ by implementing the MEAN stack (Holmes and Herber, 2019). The version of Angular that we used here was CLI 8.3.23. In addition to those technologies, we were also using NPM libraries designed to support the MEAN stack. We used JavaScript for interfacing with MongoDB, Express.js, and Node.js as well as several free packages available in NPM to support these technologies^20^. For instance, we used *Visual Studio Code* (version 1.48) as a programming platform and *Avast Secure Browser* as a testing browser. Avast provides a built-in test system for small devices such as smartphones.

### Angular

After compilation, Angular generates *Single Page Applications* (SPAs), which means that the code is sent to the browser at once when the user accesses the page for the first time. The main benefit of this approach is to create *dynamic pages*, improving the navigation experience to the frontend user. Angular speeds up the server–client communication by avoiding multiple client accesses and enabling complex calculations as well as data validations within the client browser. Moreover, the main difference of SPAs compared to a classic web application based on PHP (i.e., *static pages*) is that it does not load the page when one changes from page to page since all the code is already on the browser. Therefore, the main benefits of Angular are that (i) heavy calculations can be performed on the frontend side, which can alleviate the computing charge on the server; (ii) pre-validated data may be submitted to the server, avoiding the need for *back and forth* validation process; (iii) TypeScript (a superset of JavaScript) has the structures of a conventional programming language with powerful build-in libraries (e.g., RxJS), which enables the performance of scientific calculations on the frontend side if needed.

We also took advantage from the Angular library called *Angular Material^21^*, which allows predefined functions such as forms and themes. Angular Material can be used either within the HTML language as predefined tags or within TypeScript for dynamic pages (e.g., for Reactive forms). We used Angular Material within TypeScript since it provides much more programming freedom, e.g., form validation.

### Node.js

One of the key features of Node.js is that it allows the usage of JavaScript (or TypeScript) on the server-side. Until then, JavaScript was restricted to browsers and this progress has been possible due to the V8 Engine that compiles JavaScript code to native machine code at runtime. We used the NPM repository to install and manage all the Node.js (version 10.16.3) packages.

Node.js applications are *stateless*, which means that they do not keep information about the user stored locally and for that reason only require low amount of local RAM. Node.js applications are also single thread, which means that they do not stop the main thread as they result from users’ interactions.

We chose the *JSON Web Token* (JWT) approach to save the user information temporally on the frontend. JWT is an encoded string used when the frontend communicates with the server. The benefits of JWT are (i) that it carries a server signature, which must match whenever the user tries to communicate with the server, and (ii) that an expiration date may be set, which implies token refreshing.

### Express.js

Express.js is a library whose purpose is to facilitate the exploration of the Node.js functionalities (e.g., creating routes and servers). Here, we used Passport.js^22^ together with Express.js (version 4.16.1) to build user sections as described by Holmes and Herber (2019).

### MongoDB

MongoDB can be accessed through Angular using Node.js as server; Angular serves as a frontend framework for users (Fain and Moiseev, 2018). MongoDB is *horizontally expandable^23^*, which enables to expand storage capability without extensive physical changes. This feature decreases the cost of prototyping and posterior expansion. Another interesting property of MongoDB is the *MongoDB Atlas^24^*, which provides cloud storage.

The usage of MongoDB with Node.js is facilitated by the Mongoose^25^ library. Mongoose, amongst other benefits, allows (i) the usage of JavaScript as a programming language, which saves the need for database programming, (ii) the modeling of data before their storage into MongoDB, and (iii) the easier exploration of the MongoDB horizontal scaling capability^26^.

### Angular Flex-Layout

According to Fain and Moiseev (2018), we used a single code to implement *Responsive Web Design* (RWD) to optimize maintenance costs. This strategy allows the user interface layout to change in response to the device screen size (desktop or cell phone). RWD allows the interface simplification on small devices by limiting the display of extra-small devices to key functions (see Supplementary Figure 1 for screen size and Angular screen size settings).

We tested the responsiveness of our portal on a desktop computer using the built-in developer tool of Avast Secure Browser. We also tested it on the following devices: Moto G4, Galaxy S5, Pixel 2, Pixel 2 XL, iPhone 5/SE, iPhone 6/7/8, iPhone 6/7/8 Plus, iPhone X, iPad, iPad Pro. However, the Avast Secure Browser simulator does not necessarily consider the operating system, and it may give an unexpected display in uncommon devices.

### Passport.js

For creating the user section, we used Passport.js^27^. Its main benefits are the possibility of (i) creating customized login system or use pre-defined ones, such as those of Facebook, for example; and (ii) using it with JWT tokens due to their built-in libraries that facilitate their use. To implement JWT within Passport.js, we used *express-jwt^28^*, which allows the validation of JWT tokens, including expiration date and abnormal tokens.

### Forms

The function of the patient main form is to collect and to store basic information regarding the patient and its tissue samples for genetic analysis. This information is necessary for the posterior retrieval from the system database of patients’ medical records. Patient data are central to the system since they articulate genetic analyses with medical records that must be encrypted (e.g., patient name, mother’s name, and patient id). The patient data collected through the main form of the frontend are stored together with genetic data from the backend within MongoDB.

The request for a genetic exam is of key importance when it comes to the service provided. When physicians send tumor samples, they will be asked to request their gene expression analysis and provide patient information as well as medical records (see Supplementary Figure 2).

The outcome form has such as (i) details of the treatment applied, (ii) treatment benefits, (iii) whether the gene expression-based recommendations were followed, and so forth (see Supplementary Figure 3). The outcome form is essential for establishing case statistics.

Angular provides two options when it comes to forms: *Template-Driven Forms* and *Reactive Forms^29^*; we used the latter. The main reason for this choice was that this option provides (i) a set of built-in routines for form validation, including error messages that can easily be shown on the frontend, and (ii) the possibility of building its own customized error handling routines. By error, we mean any input to the form fields that does not fit what is expected, e.g., e-mail out of the format or password that does not match. We were also using form validators that communicate with the server on the background side to check data consistency.

Additionally, we used FormBuilder^30^ that is an Angular service used for the programming of Reactive form. With FormBuilder, one can construct JSON objects (our data format), validate the inputs of the forms individually or as a group, and other functionalities.

### Encryption, Decryption, Hashing, and JWT Coding

Since we are dealing with potentially sensitive information, we followed standard practices to protect the information submitted to the system and stored on our database. In the current stage of development, we are using standard libraries, which can be replaced by more secure ones as soon as the platform scale up. In the current version, we are using three different approaches to protect information from potential unauthorized accesses: (i) encryption/decryption, (ii) hashing, and (iii) JWT (e.g., communication with API^31^). For encryption/decryption, we are using the library *CryptoJS*.^32^ The ‘secret’ is kept on the server using a library known as *dotenv^33^*, which is largely used to store sensitive information in Node.js applications. For hashing, we are using the library *bcrypt^34^* in the following configurations: *bcrypt.genSalt*(*10, callback*), the first argument is the size of the *salt* and the second is the function for hashing.

The code for the web site can be downloaded from GitHub: https://github.com/Teranostico under the MIT License.

## Results

### Galaxy Pipeline

**FIGURE 2:**
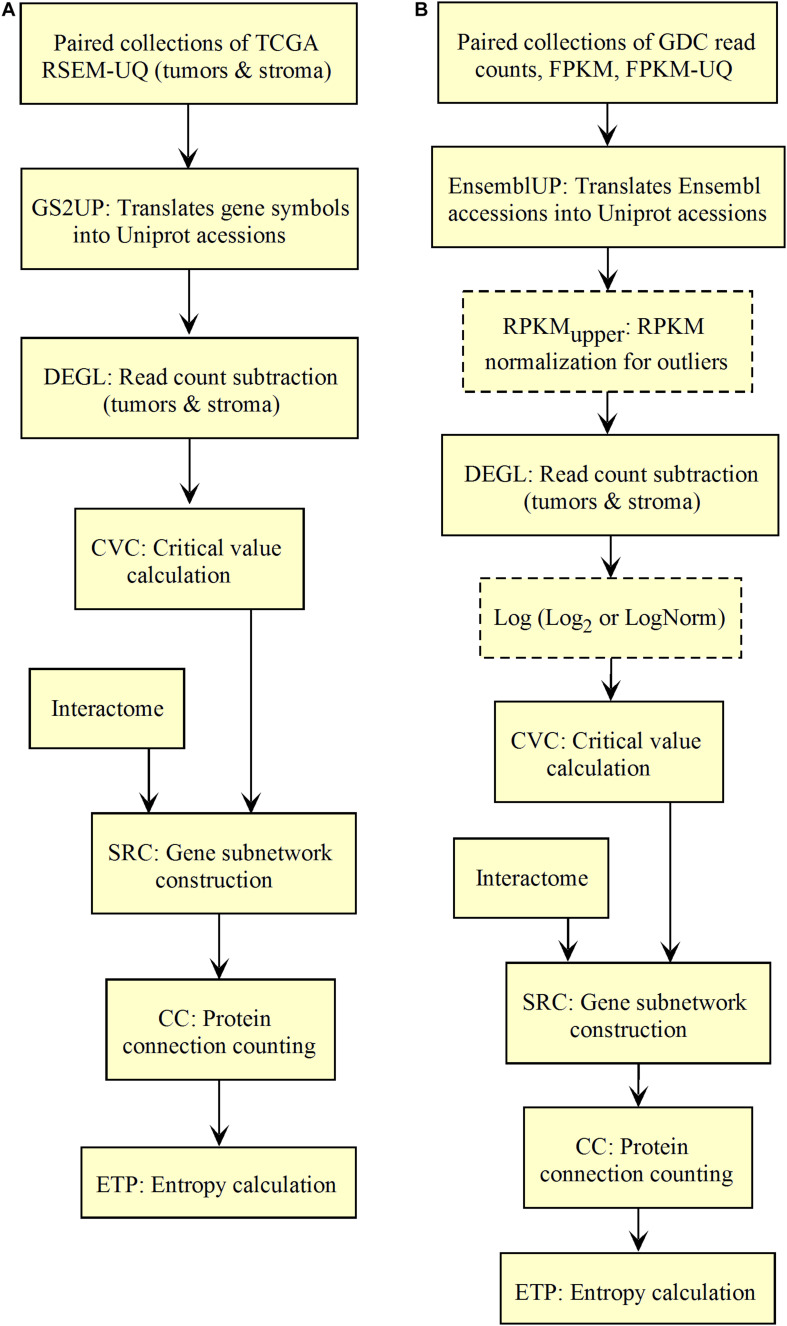
Workflow for the validation of the correlation between the entropy of the subnetworks of up-regulated genes from different tumors and their respective 5-years OS. **(A)** TCGA. **(B)** GDC.

We validated and automated the process published by Conforte et al. (2019). Thus, one sought to reproduce the results obtained by Conforte et al. (2019) when the pipeline was fed with the same data (TCGA RSEM-UQ). We indeed succeeded to reproduce the correlation *r* = –0.68 between entropy and patient’s 5-years OS for a probability of *p* = 0.975 in the determination of up-regulated genes, which allowed us to test whether the maximization of *r* really occurred for *p* = 0.975. To meet this challenge, we measured the correlation coefficient for *p* = 0.97 and *p* = 0.98, and found *r* = –0.53 and *r* = –0.60, respectively. The automated workflow is given in Figure 2A.

As shown in Figure 2A, the *input data collection* represents a collection of paired samples (tumors identified as 01A and control identified as 11A) with the same list of genes (identified by gene symbol) for each patient of the TCGA database. Following the processing flux, the gene symbols are transformed into UniprotKB accession numbers (GS2UP) to perform the subtraction of the control RNA-seq expression data from that of the tumor (DEGL). The calculation of the critical value that identifies up-regulated genes is performed by the Python script CVC. The critical value is calculated according to a probability level chosen by the user and is used by the script SRC for extracting the list of up-regulated genes. This list is used by the CC script for counting the connections at each vertex of the subnetwork of up-regulated genes. The connection count at each vertex is necessary for computing the Shannon entropy of the tumor subnetwork of up-regulated genes by the ETP script.

We validated the pipeline with the GDC *raw counts* comparing their RPKM_upper_ to the TCGA RSEM-UQ (Figure 2B without the log transformation step). First, we computed the *raw counts* according to RPKM_upper_ excluding BRCA and LUAD because of inconsistencies between file names available for FPKM-UQ and *raw counts*. In both BRCA and LUAD, cleaning samples for perfectly matched files led to sample size below *n* = 20, which may bias comparison (sample size is considered to be statistically trustworthy from at least *n* = 30 and needs correction below this threshold). When we compared the critical values for *p* = 0.975 considering the *raw counts* normalized with RPKM_upper_ (Table 2, column GDC RPKM_upper_), we found values similar to those obtained by processing TCGA RSEM-UQ data (Table 2, column TCGA RSEM-UQ).

**TABLE 2:** Critical values of probability density for *p* = 0.975.

Cancer	GDC RPKM_upper_		TCGA RSEM-UQ		GDC RPKM_upper_ + LogNorm		GDC FPKM-UQ	
Type	Av.	StDev	Av.	StDev	Av.	StDev	Av.	StDev
PRAD	2661.73	498.89	2566.88	507.38	15558.79	1053.56	15809.77	779.91
LUAD	2897.95	437.50	3138.07	313.74	15720.85	1221.94	16340.59	860.48
LUSC	3532.06	426.30	3527.89	429.98	15775.55	857.31	16161.27	730.71
BRCA	3211.72	434.50	3024.87	465.83	15346.96	664.95	15923.64	682.80
KIRC	3133.16	236.39	3162.20	363.44	15820.34	742.76	16310.36	604.57
KIRP	3084.69	365.17	3089.64	390.28	15482.35	905.77	16165.59	597.30
THCA	2610.75	313.49	2590.59	406.12	14876.38	1089.35	15559.35	713.54
STAD	3330.13	444.58	3273.89	470.64	16511.00	865.11	16473.27	718.44
LIHC	3085.76	474.40	3409.36	468.48	16235.74	1087.23	15639.15	801.43
Average	3060.88	403.47	3139.90	420.79	15703.11	943.11	16042.55	721.02
St. Dev.	298.36	83.63	299.07	56.29	479.01	181.82	324.01	86.51

We found that critical values for *p* = 0.975 of GDC FPKM-UQ were ∼5 times larger (Table 2, column GDC FPKM-UQ), on the average (Figure 2B without normalization and log transformation steps), than those of TCGA RSEM-UQ (Table 2, column TCGA RSEM-UQ and GDC RPKM_upper_). This difference is due to the processing update performed during the data transfer from TCGA to GDC portal involving the flattening of the differential gene expression distribution.

When we successively computed GDC *raw counts* with formula 6 (RPKM_upper_) and 7 (LogNorm), we found critical values for *p* = 0.975 (Table 2, column GDC RPKM_upper_ + LogNorm) close to that of GDC FPKM-UQ (Table 2, column GDC FPKM-UQ), suggesting a similar behavior of differential gene expression flattening as the one applied by the GDC data processing (Figure 2B).

The comparison of the size of subnetworks of up-regulated genes in tumors is given in Table 3. The difference of subnetwork size between GDC FPKM-UQ and GDC RPKM_upper_ + LogNorm samples, on one hand, and TCGA RSEM-UQ and GDC RPKM_upper_ samples, on the other hand, raised the question of whether the large subnetwork size of GDC FPKM-UQ and GDC RPKM_upper_ + LogNorm might be trusted.

**TABLE 3:** Size of subnetwork (vertex number) of genes up-regulated in tumors for a probability density of *p* = 0.975.

Cancer	GDC RPKM_upper_		TCGA RSEM-UQ		GDC RPKM_upper_ + LogNorm		GDC FPKM-UQ	
Type	Av.	StDv.	Av.	StDv	Av.	StDv.	Av.	StDv.
PRAD	269.19	62.01	254.20	40.66	5046.23	1209.45	4029.75	499.89
LUAD	290.35	58.66	276.35	49.07	4973.16	1203.25	4779.27	401.19
LUSC	345.21	48.50	317.12	48.33	5824.63	904.38	4981.60	460.49
BRCA	311.55	46.50	286.50	42.16	5305.85	1219.24	4816.61	361.22
KIRC	332.28	42.37	328.10	52.85	5117.83	881.77	4556.75	294.80
KIRP	313.48	49.64	303.22	41.14	4983.68	1136.93	4678.77	305.26
THCA	256.52	47.31	276.95	57.44	4016.13	948.02	4142.73	387.09
STAD	341.67	52.90	276.59	51.66	6773.41	928.62	4764.48	351.76
LIHC	352.74	68.99	256.24	85.31	7007.28	143.05	4522.08	400.83
Average	312.55	52.99	286.14	52.07	5449.80	1096.08	4585.78	384.72
St. Dev.	34.34	8.57	25.49	13.72	942.86	189.53	315.90	66.69

The subnetwork sizes obtained by successively processing GDC *raw counts* with formula 6 and 8 (Table 4, column Node number) were smaller and more realistic, representing between ∼2% and ∼5% of the human proteome.

**TABLE 4:** Critical values of RPKM_upper_ + Log2 for a probability density of *p* = 0.975 and vertex number of subnetworks of genes up-regulated in tumors.

Cancer	Critical value		Vertex number	
Type	Average	StDev	Average	StDev
PRAD	7359.58	1019.74	884.69	248.58
LUAD	7985.00	1105.05	946.58	219.40
LUSC	9325.45	1187.62	1244,35	232,65
BRCA	8398.81	1232.49	1087.20	240.74
KIRC	8335.51	561.98	1035.82	143.58
KIRP	8299.87	777.86	1014.35	206.89
THCA	7210.95	706.19	775.96	140.05
STAD	9173.28	1154.74	1264.93	249.58
LIHC	8398.81	1232.49	1235.50	294.36
Average	8276.36	997.58	1054.38	219.53
St. Dev.	707.07	251.53	171.09	50.27

As explained above, we did not consider BRCA and LUAD for comparison between RPKM_upper_ and FPKM-UQ. However, the FPKM-UQ correlation plot was similar to that of other authors (data not shown).

The features of the linear regression between the subnetwork entropies and the 5-years OS are given in Table 5 for the different pipeline configurations tested here.

**TABLE 5:** The features of the linear regression between the entropy and the 5-years OS for *p* = 0.975.

Normalization method	Coef. Correl. (with BRCA + LUAD)	Coef. Correl. (without BRCA + LUAD)	Regression (without BRCA + LUAD)
GDC RPKM	–0.36	–0.55	–
GDC RPKM_upper_	–0.68	–0.86 (Figure 3A)	*y* = –0.0084x + 2.717
GDC RPKM_upper_ + LogNorm	–0.67	–0.85 (Figure 3B)	*y* = –0.0090x + 4.473
GDC RPKM_upper_ + Log2	–0.69	–0.91 (Figure 3C)	*y* = –0.0096x + 3.460
GDC FPKM	–0.11	–0.13	–
TCGA FPKM-UQ*	–0.68	–0.64	*y* = –0.004x + 2.507
GDC FPKM-UQ	–0.71	–0.76 (Figure 3D)	*y* = –0.0039x + 4.025

*See Conforte et al., 2019.

Interestingly all the combinations involving RPKM_upper_ of Table 5 resulted in a larger slope of the regression line; in other word, they resulted in an increased statistical significance of the regression line.

**FIGURE 3:**
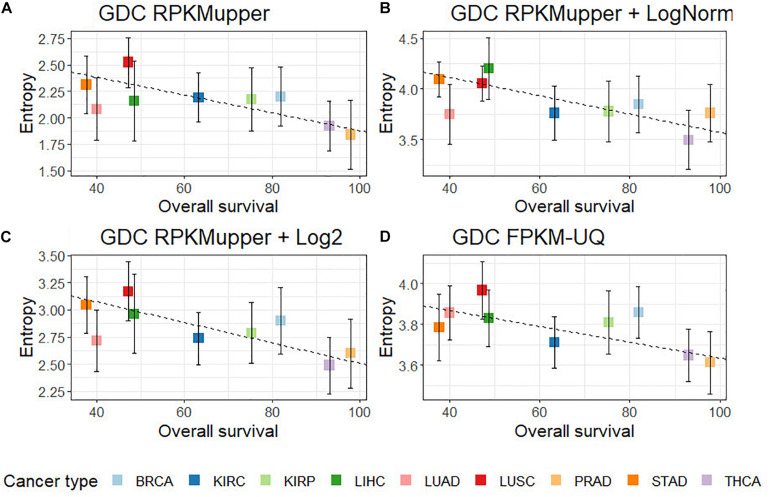
Correlation of subnetwork entropies vs. 5-years OS for *p* = 0.975. **(A)** GDC RPKM_upper_. **(B)** GDC RPKM_upper_ + LogNorm. **(C)** GDC RPKM_upper_ + Log2. **(D)** GDC FPKM-UQ.

Compared to GDC RPKM_upper_ (Figure 3A), the introduction of the LogNorm in the workflow of Figure 2B resulted in a systematic shift of entropies by as much as ∼1.5 bit toward larger values (in the range of 3.6–4.0 compared to 2.0–2.5 in Conforte et al., 2019), which denote a larger subnetwork of up-regulated genes with larger number of hubs as a consequence of the distribution flattening of differential gene expression. The correlation obtained by successively processing *raw counts* with RPKM_upper_ and LogNorm (Figure 3B) was similar (*r* = –0.86 without BRCA and LUAD) to that obtained with GDC FPKM-UQ (*r* = –0.76 without BRCA and LUAD) (Figure 3D). Finally, it is the correlation obtained by successively processing *raw counts* with formula 6 and 8 (Figure 3C) that showed the best correlation coefficient and slope of the regression line (*r* = –0.91).

**FIGURE 4:**
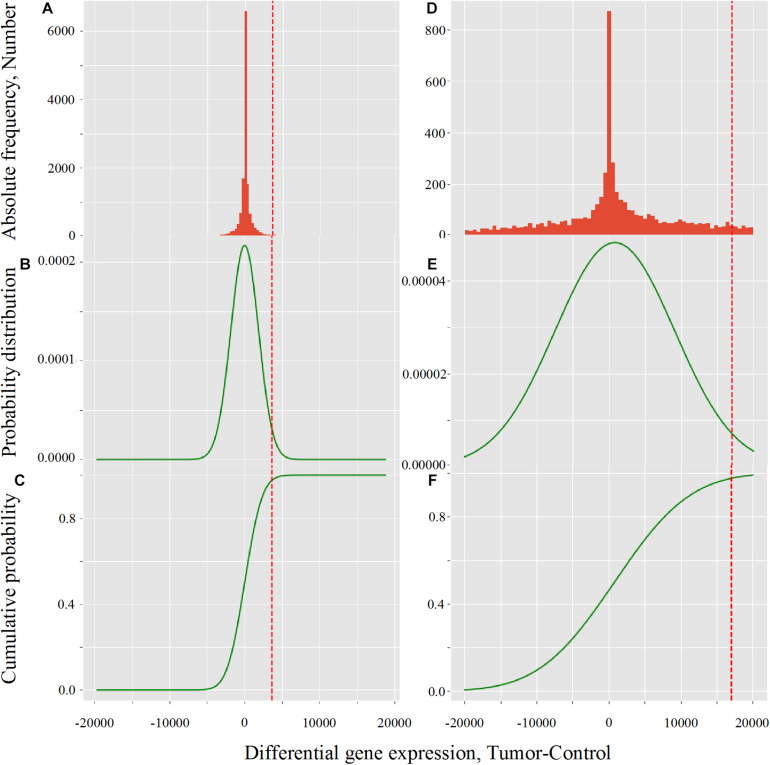
Critical value calculation (red dot line) by CVC script in TCGA **(A–C)** and GDC **(D–F)** in LUSC (TCGA-22-4593 sample). **(A,D)** Histogram of observed differential gene expression distribution (tumor-control) of genes. **(B,E)** Function of density of probability. **(C,F)** Function of cumulated probability. The critical values were 3,633.8 and 17,042.9 for TCGA and GDC, respectively.

The effect of LogNorm on distribution flattening of differential gene expression when comparing RPKM_upper_ to RPKM_upper_ + LogNorm was similar to that observed when comparing TCGA RSEM-UQ (Figure 4A) to GDC FPKM-UQ (Figure 4D), respectively.

When we compared the correlation coefficient according to *p* for GDC FPKM-UQ data, we obtained *r* = –0.758, *r* = 0.763, and *r* = 0.477 for *p* = 0.95, *p* = 0.98, and *p* = 0.99, respectively. This result shows that the maximum of *r* was associated with *p* = 0.98, but the difference with *p* = 0.975 was only 0.002 units of the correlation coefficient, which confirmed that the peak around the maximum of *r* was less sharp for GDC FPKM-UQ than for TCGA RSEM-UQ since it spreads over *p* = 0.95 and *p* = 0.98.

**FIGURE 5:**
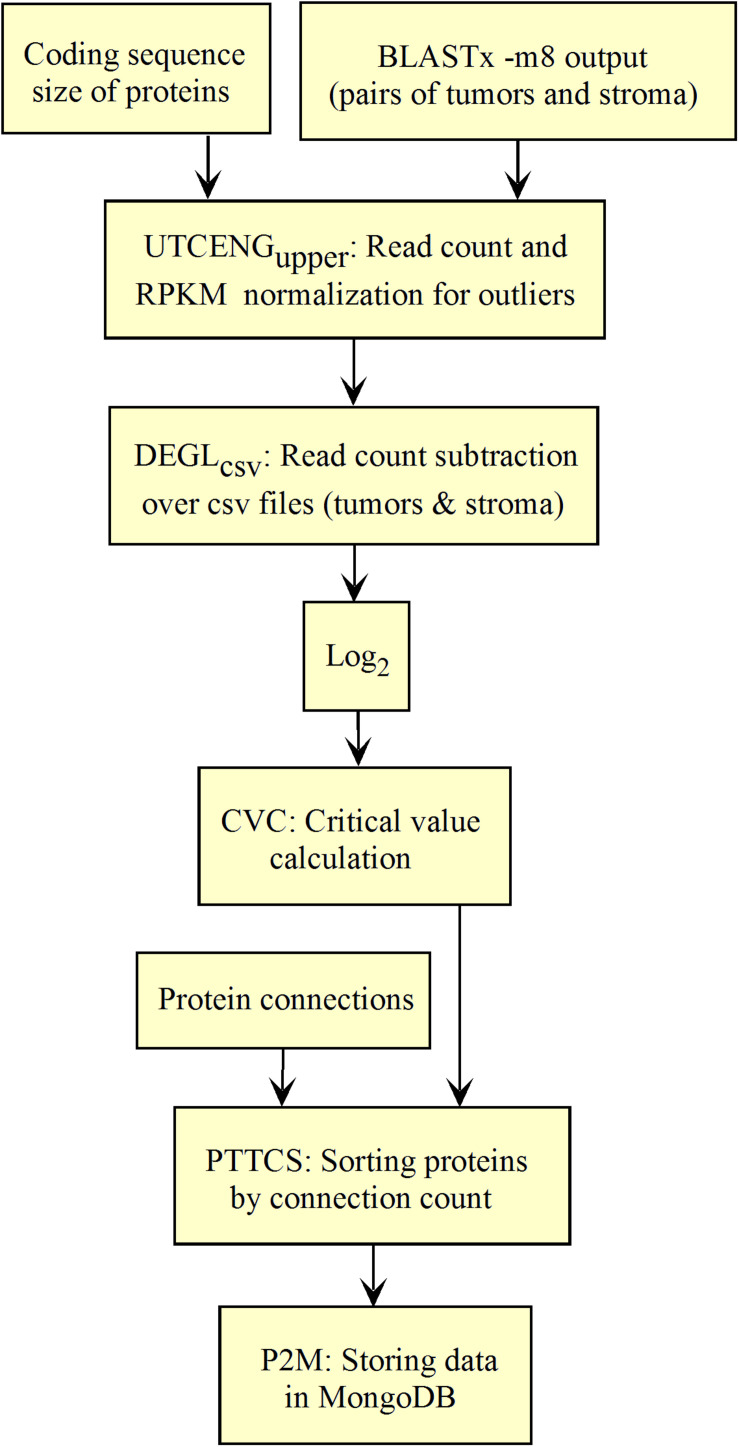
Workflow for collections of BLASTx output processing.

The flattening of the correlation peak according to the probability density appeared as a consequence of the probability density distribution shape. The distribution of FPKM-UQ values was flatter in GDC FPKM-UQ (Figures 4D–F) compared to TCGA RSEM-UQ (Figures 4A–C), which is reflected by larger critical values associated with GDC (Table 2). The validation of the mapping process of reads on the EBI interactome proteins needed similarity comparison of *fastq* files using BLASTx. We performed this validation by recycling the components of Figure 2 for processing RNA-seq data as shown in Figure 5.

The workflow shown in Figure 5 needed to be fed with BLASTx outputs. After mapping reads to their respective protein sequences in the interactome, both tumor and control *raw count* files were normalized (UTCENG_upper_) according to their coding sequence size (RPKM_upper_ step) and expression level using formula 2. The rest of the pipeline is as in Figure 2 except for the last step of sorting by decreasing level of connection (PTTCS) and data storage in MongoDB (P2M).

The list of top-n connected up-regulated hubs is released as output data from the workflow, and stored in MongoDB (Figure 5) together with the patient’s clinical data. These data can be formatted as a medical report by the JavaScript code within the web page according to the user request.

Considering the entropies of subnetworks of NSCLC up-regulated genes (*x*_1_ = PRJNA320473) and PRAD (*x*_2_ = PRJEB2449), the *u*_*obs*_ calculated with formula 3 with ${\bar{x}}_{1}$ = 2.99475 and ${\bar{x}}_{2}$ = 1.66472, respectively, as well as *SCE*_1_ = 10.31347 and *SCE*_2_ = 6.70566, respectively, was 5.00748. Since *k* was found to be 29.06411 (∼29) for the sample sizes considered, the theoretical values of *t* for *p* = 0.975 and *p* = 0.999 were 2.045 and 3.396, respectively. Because *u*_*obs*_ > *t*_*th*_, we rejected the null hypothesis of average equality for NSCLC and PRAD and concluded that the entropy of NSCLC was significantly larger than that of PRAD. This result is in agreement with the negative correlations of Figure 3 and validates the pipeline here presented.

As the methodology was validated, it could be used for the diagnosis of the top-n most connected proteins within the list of up-regulated genes in the tumor compared to the stroma. It is important to underline that the entropy was used only for the purpose of methodology validation.

**FIGURE 6:**
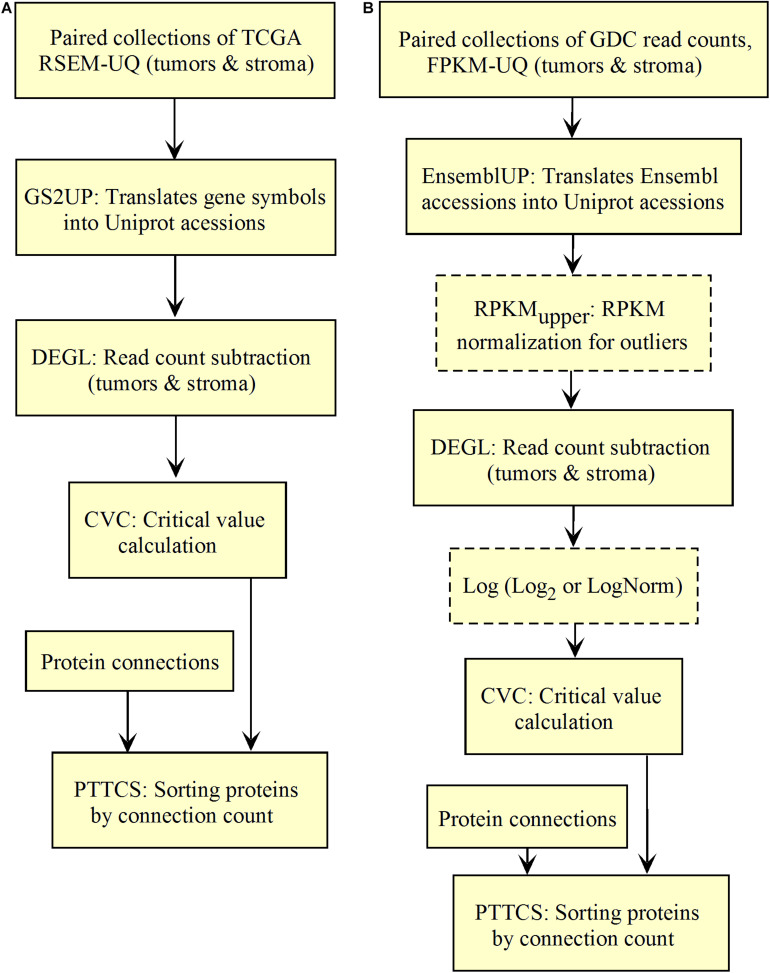
Workflow for top-n most relevant hub target diagnosis in up-regulated genes of solid tumors. **(A)** TCGA. **(B)** GDC.

A pipeline to identify the connection hubs is given in Figures 6A,B, where the purpose of PTTCS is to compare up-regulated genes to the list of vertex connections in the interactome to rank them in decreasing order of connection number in the output file. *A priori*, top-20 most connected proteins among the up-regulated genes of tumors should be enough to design a personalized treatment. However, this number depends on drug availability.

The comparison of the most relevant targets associated with the different normalization methods applied in this report is shown in Table 6. Table 6 reports the number of tissues (# column) where the gene of a given protein (Acc column) was up-regulated among nine different tumors. For illustration, we only kept genes up-regulated in at least 70% of tumor samples of each cancer type (pink). The colors in the first column report for the targets that are common between different sections (A to E) of Table 6 (turquoise is for the genes common to Tables 6A–E; blue is for the genes common to Tables 6A,B,D,E; yellow is for the genes common to Tables 6A–C,E; mallow is for the genes common to Tables 6A,B,E; and green is for the genes common to Table 6D,E).

**TABLE 6A:**
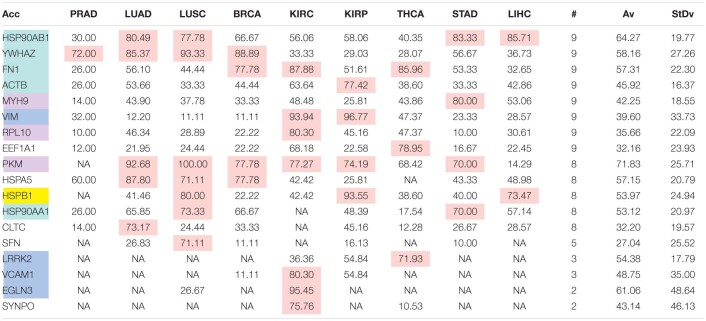
Comparative pattern of distribution for the most relevant targets among solid tumors of nine cancer types according to TCGA RSEM-UQ normalization.

*The numbers in the table represent the proportion (%) of tumors of a given cancer type that showed the gene among the top-20 most connected proteins of the subnetwork of up-regulated genes. The pink color concerns up-regulated genes in at least 70% of tumor samples of each cancer type.*

**TABLE 6B:**
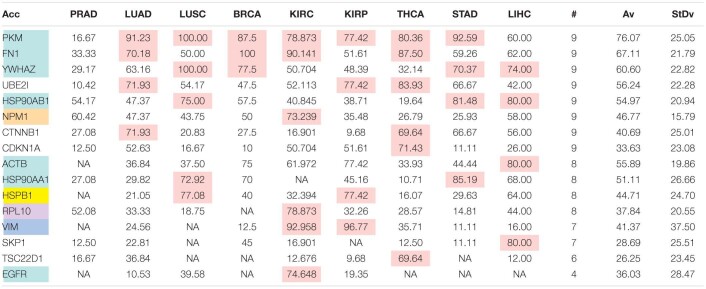
Comparative pattern of distribution for the most relevant targets among solid tumors of nine cancer types according to GDC RPKM_upper_ normalization.

*The numbers in the table represent the proportion (%) of tumors of a given cancer type that showed the gene among the top-20 most connected proteins of the subnetwork of up-regulated genes. The pink color concerns up-regulated genes in at least 70% of tumor samples of each cancer type.*

**TABLE 6C:**
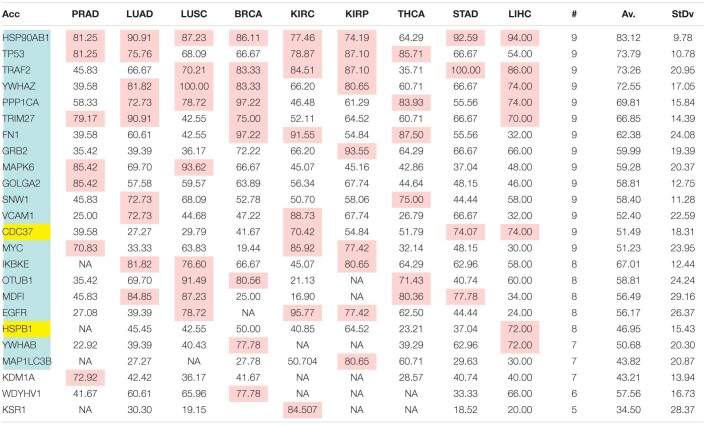
Comparative pattern of distribution for the most relevant targets among solid tumors of nine cancer types according to GDF FPKM-UQ normalization.

*The numbers in the table represent the proportion (%) of tumors of a given cancer type that showed the gene among the top-20 most connected proteins of the subnetwork of up-regulated genes. The pink color concerns up-regulated genes in at least 70% of tumor samples of each cancer type.*

**TABLE 6D:**
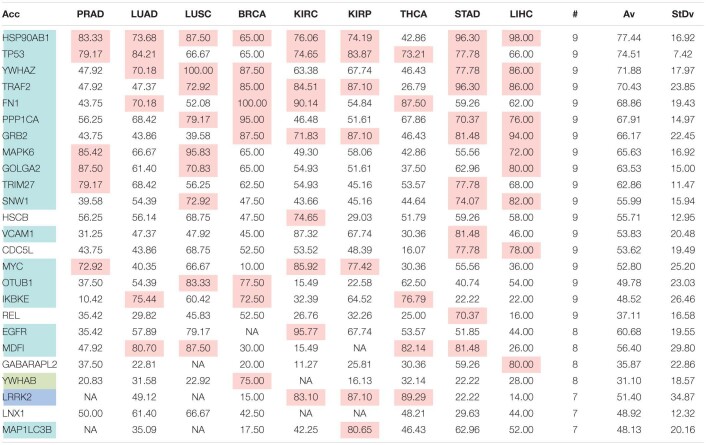
Pattern of distribution for the most relevant targets among solid tumors of nine cancer types according to successive processing through RPKM_upper_ and LogNorm normalization.

*The numbers in the table represent the proportion (%) of tumors of a given cancer type that showed the gene among the top-20 most connected proteins of the subnetwork of up-regulated genes. The pink color concerns up-regulated genes in at least 70% of tumor samples of each cancer type.*

**TABLE 6E:**
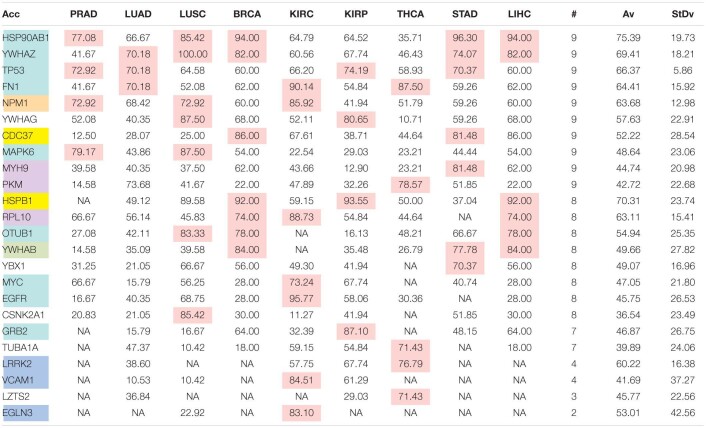
Pattern of distribution for the most relevant targets among solid tumors of nine cancer types according to successive processing through RPKM_upper_ and Log2 normalization.

*The numbers in the table represent the proportion (%) of tumors of a given cancer type that showed the gene among the top-20 most connected proteins of the subnetwork of up-regulated genes. The pink color concerns up-regulated genes in at least 70% of tumor samples of each cancer type.*

Tables 6A–E show that the most relevant targets are largely shared among methods. In Tables 6C,D, target personalization according to the tumor was lower than in Tables 6A,B,E. Because of the larger average network size that it produced, the normalization with RPKM_upper_ + Log2 (Table 6E) showed a larger targets number than TCGA RSEM-UQ and GDC RPKM_upper_ (Tables 6A,B), similar to those of Tables 6C,D but with a larger level of tumor personalization. Because of the reasonable size of subnetworks and the best correlation relationship between entropy and the 5-years OS it produced, the successive processing through RPKM_upper_ and Log2 normalization was considered here as the best compromise.

### Scaling Analysis

**FIGURE 7:**
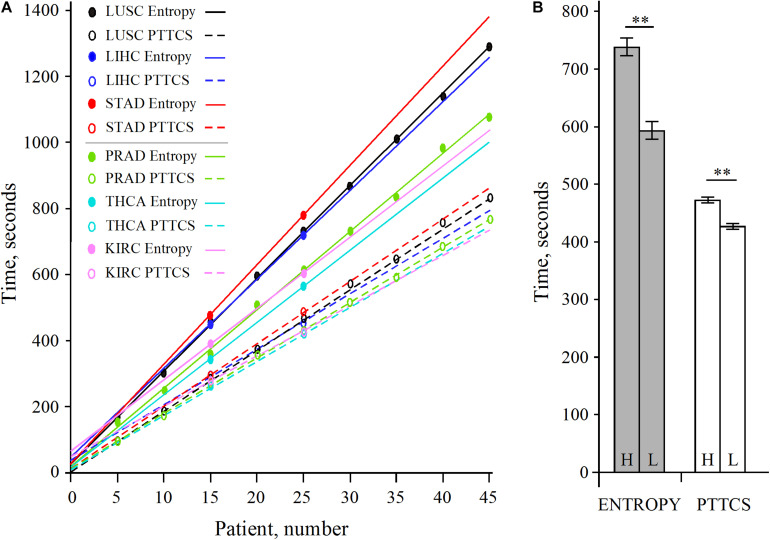
Scaling of pipeline from Figure 2B (entropy) and Figure 6B (PTTCS) using GDC read counts (see data in Supplementary Table 1). **(A)** Linear scaling for 5–45 patients in LUSC and PRAD as well as 15 and 25 patients for STAD, LIHC, THCA, and KIRC. **(B)** Statistical analysis of scaling for high entropy (H) cancer (LUSC, LIHC, STAD) and low entropy (L) cancer (KIRC, PRAD, THCA) for entropy (gray) and PTTCS (white) pipelines. ^∗∗^Significant at α ≤ 0.01 for *k* = 4 degrees of freedom. The horizontal bars are for the standard error of the mean (SEM).

The analysis of LUSC and PRAD over 45 patients showed that the scaling of pipeline processing is linear and perfectly predictable (Supplementary Table 1 and Figure 7A). In addition, Supplementary Table 1 shows that the entropy pipeline takes a systematically larger time to be completed for high entropy cancer types than for low entropy cancer types. This is also true for the hub diagnosis pipeline (PTTCS). A more careful analysis for 25 patients for LUSC, STAD, LIHC, on one hand, and PRAD, THCA, KIRC, on the other hand, showed that this assumption is statistically significant (Figure 7B). Considering the pipeline for hub diagnosis from BLASTx output, we found the time series 50, 94, 137, 187 and 53, 100, 145, 190, for PRAD and NSCLC, respectively. These differences were not significant, but suggest that this pipeline scales similarly to the PTTCS one.

### Web Application

The web application implements the graphical interface that allows the user to interact with the forms and their respective accounts (i.e., private areas). As outline above, it is the server that runs Galaxy and hosts MongoDB that stores the up-regulated hubs and patient data introduced by the user, which are necessary to produce the medical record. The frontend includes a succession of forms for data introduction and a private area, which allow access to patient data whenever necessary with user’s privileges.

### User Private Area

**FIGURE 8:**
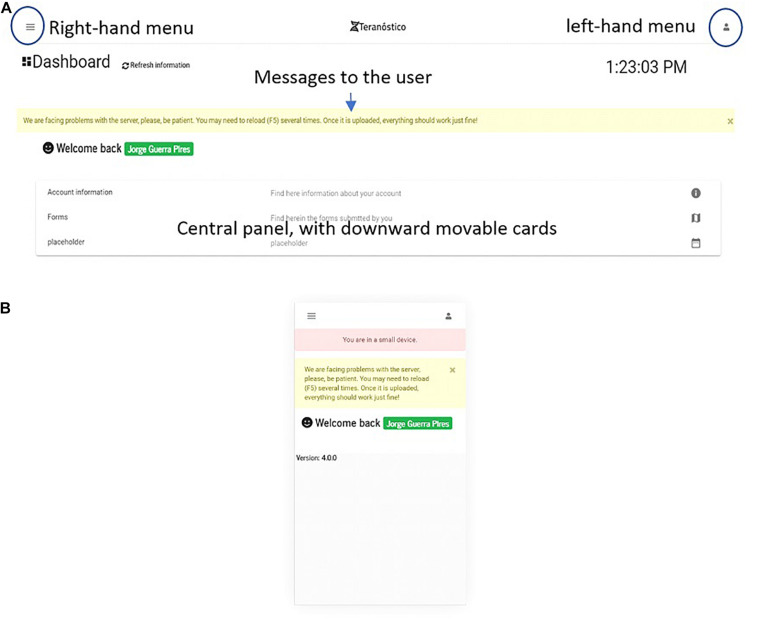
Dashboard on **(A)** a desktop and **(B)** an extra small devices (Galaxy S5).

The private area is the section accessed by the user after logging in (see the dashboard in Figure 8). The key advantages of a private area are that (i) the user may access their information any time, (ii) sections can be customized, with different levels of privileges, (iii) they can be customized according to business models (Blank and Dorf, 2012).

### Dashboard

The dashboard (Figure 8) is the first page one sees when accessing the platform after login in from the *welcome* page. On the welcome page, users can register an account. The main goal of the dashboard is gathering all the essential information contained in the portal for the logged in user (e.g., forms to be submitted by users). Thus, users can either introduce the data of their patients or retrieve analysis reports, if they are physicians or administrate the platform, if they are system administrators.

We implemented a simplified version for small devices to fit their screen size and limit the system to the essential (Figure 8A). The user is informed when using the system on small devices, which is a benefit compared to Bootstrap. As a result of screen simplification, most of the information from the desktop version (Figure 8B) is omitted on small devices, which means that users must access the platform either from desktops or middle size devices (e.g., iPads) for a full-version.

### Components

Components in Angular are a set of three types of files: CSS (appearance-related), TS (typescript, coding), and HTML (classic static page). A page is built from at least one component, which can independently interact with each other (Fain and Moiseev, 2018). From a software engineering viewpoint, this technology makes the pages more dynamic and faster, and its parts can be easily reused on other pages. The main components of the dashboard are the menu and central cards. The menu, located upward, displays basic and customized information eventually organized in options. The central cards, movable downward, display information and make them available as active links (e.g., a list of forms submitted by the user).

### Protecting Confidential and Sensitive Information

Patients’ data are confidential and require protection as stated by policies all over the world (e.g., *Health Insurance Portability and Accountability Act*, HIPAA for the United States). Thus, new users must first register and enter some basic information to gain access to the server.

### Login

The *login card* (Supplementary Figure 5) is a standard login page. In the current frontend version, we are following a simple login system strategy. Essentially, the user must enter its e-mail and password as previously registered to log in and access the dashboard.

Since we are storing JWT locally, it is up to the user to decide when to log out. Normally, JWT expires after 15 min on a standard basis; we set the expiration time to 1 day. This approach avoids repeated login whenever the JWT expires.

### Forms

In the current frontend version, we have two sets of forms: the patient main form (Supplementary Figure 7) and the outcome form (Supplementary Figure 8). The patient main form is expected to be sent alongside the patient samples, which is independent, while the outcome form is expected to be sent in case of death (for documentation).

All the information related to a patient is stored in different documents and is merged for display using a method called *populate* from Mongoose, which enable the information retrieval from other related documents.

**FIGURE 9:**
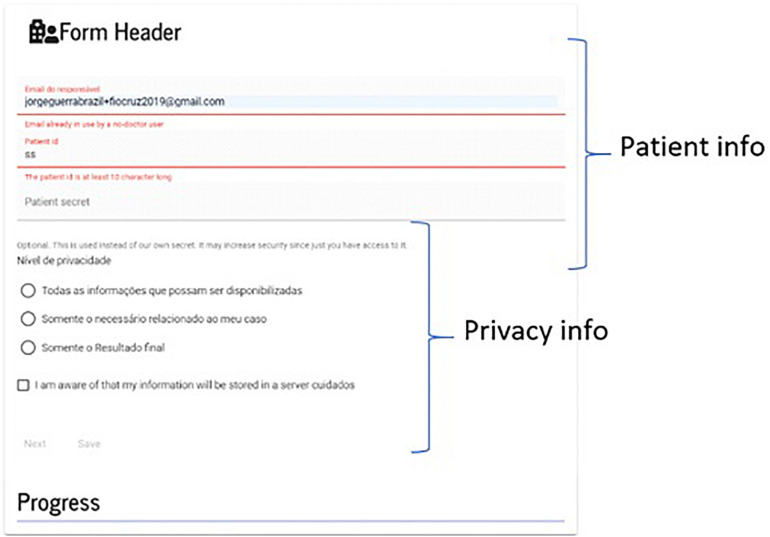
Form header. A user is warned when leaving without saving the form. The patient password is encrypted and kept on the server using a specialized type of file; nonetheless, users can choose their own passwords.

Because of this design, we created a *header* form (Figure 9), whose function is to (i) collect encrypted patient id, (ii) provide a password for encryption (optional), and (iii) provide privacy-related options.

The form remains in contact with the server for validating information on the background, while the user is filling out the fields; most of the validations are done without communication with the server.

Sensitive information are entered on the first page and encrypted in a similar way to the data introduced through the header. Any form can be recovered from a list of links that are made available on the *movable card* on the dashboard.

Finally, a submission receipt is automatically generated upon form submission (see Supplementary Figure 9), which provides the user with the information necessary for future access to the forms submitted (see Supplementary Figure 10).

## Discussion

### Galaxy Pipeline

In this report, we presented a workflow for processing RNA-seq data that allows the rational diagnosis of top connected hubs among genes that are up-regulated in tumors according to the non-tumoral peripheral area (stroma). The use of the stroma as a control to measure the malignant differential expression via RNA-seq has been recognized to be equivalent to the use of healthy tissues for this purpose (Finak et al., 2006). Of course, many factors may promote cancer such as chemicals, radiation as well as genetic defects in reparation and replication molecular machinery. To gain inside into such a complex problem as a molecular approach of cancer together with a still-evolving protocol of RNA-seq treatment regarding normalization procedure or error rate (Li et al., 2020), a robust measure was needed. We found this measure in the degree entropy. Entropy offers the benefit to be independent of sample size. In this report, we calibrated our approach by reference to OS, but after an optimization round for the treatment of RNA-seq data, other factors could be taken into account to understand how they interact with the signaling network complexity.

Normalization of raw read counts account for (i) within-sample effects induced by factors such as coding sequence size (Oshlack and Wakefield, 2009), GC-content (Risso et al., 2011), (ii) between-sample effects such as sequencing depth (total number of molecules sequenced) (Robinson and Oshlack, 2010), and (iii) batch effect (Tom et al., 2017). As underlined by Evans et al. (2018), “normalization methods perform poorly when their assumptions are violated.” Thus, the exercise is to “select a normalization method with assumptions that are met and that produces a meaningful measure of expression for the given experiment.”

Following these recommendations, we must first consider that the purpose of our approach is to list the top-n most relevant target among subnetworks of genes that are up-regulated in tumor samples compared to their controls. Consequently, the complexity of the up-regulated gene subnetwork must be coherent with the 5-years OS. Indeed, our supporting hypothesis is that the complexity of the malignant subnetwork or the number of times that the malignant subnetwork can reorganize itself after perturbation is in line with its information content, i.e., its Shannon entropy. This is the reason why it makes sense to optimize the normalization process for maximizing the coefficient of correlation between entropy and 5-years OS. We aimed to diagnose the subnetwork complexity because it is correlated to the 5-years OS and this is important for therapy’s success (whatever being performed with drugs or biopharmaceuticals) in the context of a personalized approach of oncology.

The PDF and CDF functions of the Python’s scipy package allowed the calculation of the critical values given the density of probability of non-differentially expressed genes. These distribution are rather similar regardless of the RNA-seq considered for a given normalization process. These genes are thousands while the up-regulated ones are hundreds, which makes critical value determined in this way rather precise and reproducible. Concerning the statistical significance of the method we applied, one has to say that we face a classification problem. In such circumstances, one usually looks for the optimization between false positive and false negative rates. However, when dealing with medical purpose, one has to look to bias the classification process toward the minimization of false-positive rate to reduce toxic drug collateral effects to patients that would derive from hubs still expressed at a significant level in the stroma (this consideration does not concern drug toxicity due to off-target effects). There is a compromise between minimizing the false positive rate and the availability of hub targets for therapy. A larger *p*-value (*p* > 0.025) would release a larger list of up-regulated genes with more hub targets; a larger list of potential drugs for the case under consideration, but also a larger probability of toxic effects on the stroma. In contrast, lower *p*-value (*p* < 0.025) will minimize toxic effect of therapy to patient, but would also decrease the number of potential hubs for therapy. Of course, this consideration neglects the tissue specific expression of genes and a gene that is up-regulated in a tumor compared to its stroma could also be up-regulated in another tissue, on a normal basis. Here, we neglected this issue, but it is possible to preferentially target tumors through nanoparticle therapy or by local application.

As pointed out by Abbas-Aghababazadeh et al. (2018), it is possible that some of the estimated *latent factors* are not technical artifacts but rather represent true biological features reflected in the data. The correction of these latent factors may introduce unwanted biases. Here, we did not want to stabilize the subnetwork size variance (Smyth, 2004; Cloonan et al., 2008; Love et al., 2014; Holmes and Huber, 2019) because we believe that it is part of the challenge. One cannot exclude the possibility of network size varying among samples according to the specificities of genome deregulation proper to a given tumor. Despite commonalities that were recognized between tumors of the same cancer type, many features such as gene demethylation, copy numbers, somatic crossing over, and chromosome karyotype contribute to the specificity of the molecular phenotype of a tumor and it is the correct diagnosis of these specificities that can make the difference in terms of patient benefits (Duesberg et al., 2005; Ozery-Flato et al., 2011; Ogino et al., 2012; Grade et al., 2015; Bloomfield and Duesberg, 2016; Ye et al., 2018; Xia et al., 2019).

According to the considerations just outlined, the size of the malignant subnetwork is also important because it directly affects the number of targets available for therapy. The size of the malignant subnetworks also depends on the normalization process. There is a tradeoff between the size of the malignant subnetwork and the level of tumor personalization that is effectively reported by the top-n targets as a result of the normalization process. From our perspective, the normalization corresponding to GDC FPKM-UQ and RPKM_upper_ + LogNorm generate subnetworks that are too large since they represent as much as 20% of the human proteome (>4,000 genes). By contrast, subnetworks produced by GDC RPKM_upper_ + Log2 normalization account for between 2 and 5% of the human proteome, which seems to be more realistic (Danielsson et al., 2013; Malvia et al., 2019).

The target lists that we found with the various normalization methods presented here were consistent among one another and with that of Conforte et al. (2019). The normalization method corresponding to the best compromise according to subnetwork size, correlation, and the target list was RPKM_upper_ + Log2, and it is that method that was, therefore, kept for new sample analyses.

To be coherent with former studies, we included LUAD and BRCA, however, these two cancer types discredited the analyses for two obvious reasons: (i) In the case of LUAD, the samples of *raw counts* did not match those of FPKM-UQ, which prohibit direct comparison between both datasets and raised the question why FPKM-UQ normalization was not performed on a large proportion of *raw counts* files and why, on the other hand, other samples were taken into account in the FPKM-UQ processing. This discrepancy may explain why LUAD does not match the regression line in GDC RPKM_upper_ + Log2, while it does in GDC FPKM-UQ; (ii) In the case of BRCA, after filtrating samples for matching between *raw counts* and FPKM-UQ, the total sample size was less than 20, which is not sufficient for statistical significance given subtype heterogeneity. BRCA is composed of four subtypes whose 5-years OS varies between 70 and 82%. Figure 3 shows that depending on sampling, BRCA could very well match the linear regression.

The relevance of inhibiting hub of connections has been proven mathematically by Albert et al. (2000) and its benefit for patients has been confirmed by Conforte et al. (2019) through Shannon entropy analysis. The negative correlation found between the subnetwork entropy and the 5-years OS is in agreement with the results obtained later on from the modeling of basins of attraction in BC with Hopfield network (Conforte et al., 2020). This study revealed that five tumor samples converged toward the basin of attraction associated with control samples instead of the tumor ones. Those samples were associated with a good prognosis, initial stages of tumor development, and four of them presented the smallest subnetwork entropy among the dataset of 70 tumor samples under study.

As the research concept has been validated through different approaches, the workflow presented here was built with the aim of automating the analysis, which will allow its translation to the medical context. With that concern, the larger time needed for entropy and PTTCS pipelines to be completed when analyzing high entropy cancer types compared to the processing time spent with low entropy cancer types suggests a positive relationship between subnetwork complexity and their processing time. If confirmed, this observation means that the computation model, presented here, reproduces a main biological feature of cancer that is the larger complexity associated with subnetwork of up-regulated genes in aggressive tumors. In any case, the difference in the processing time of the PTTCS pipeline for high and low entropy cancers was not large (∼50 s for 25 patients).

We believe that our strategy will contribute constructively to cancer treatment because the molecular phenotype of a cell is directly connected to its genetic alterations, which is not necessarily the case for genomic alterations. Genomic alterations allow a diagnosis based on probabilistic data obtained with large patient cohorts. By contrast, the molecular phenotype portraits the cell or the genomic disease and points to proteins that should be targeted in the first instance to disrupt malignant phenotypes while affecting the healthy one the least possible.

The phenotype approach also reflects which genes that malign cells most need to maintain themselves in the tissue given its selective constraints. In any pathogenic relationship, one distinguishes between *primary* and *secondary determinants* of the disease (Yoder, 1980). The primary determinants are those that make the relationship compatible (qualitative) and the secondary determinants are those that deal with its quantitative expression (virulence). Thus, the question to deal with, in the case of cancer, is to target primary determinants. When considering gene expression, one may reason that the heterogeneity is something related to secondary determinants (it is not because a cell is mutating that the new mutations are worse than the previous ones). Actually, it has been well described that a tumor developed by the accumulation of mutations in a small number of key oncogenes or suppressor genes in stem cells and that the probability of this event to occur is very low (Hornsby et al., 2007; Belikov, 2017). Thus, there is a difference between these primary mutations that allow the tumor to establish itself and the secondary ones that may affect its aggressiveness. On the same line, when one sequences the mRNA of a tumor area, one takes the gene expression profile of many cells into account. By consequence, secondary mutations promoting or inhibiting a given gene in different cell lineages inside the same tumor compensate themselves. By contrast, those genes that are key to maintain a malignant cell line will be positively selected to remain up-regulated in most cells and, therefore, if one detects a gene that is up-regulated in a tumor by comparing its expression level with the surrounding stroma, it means that it is essential for malign cell survival.

Considering the number of hubs to target, the results obtained by Conforte et al. (2019) suggest 3–10, on average. Other authors already suggested such complex mixes (Calzolari et al., 2007, 2008; Preissner et al., 2012; Hu et al., 2016; Antolin et al., 2016; Lu et al., 2017). Three to ten specific drugs may appear a small number to control such a complex disease as cancer, but the cell death induction may be explained by a cascading effect, which is larger when targeting hubs as suggested before (Carels et al., 2015a; Barabási, 2016; Tilli et al., 2016; Conforte et al., 2019). According to Conforte et al. (2019), this cascading effect would be inversely proportional to the tumor aggressiveness. The pitfall is that the number of specific drugs for hub targets that are approved by FDA is still very small (Antolin et al., 2016). While new drugs and biopharmaceuticals or products of other strategies continuously appear, key targets remain the same. Some are highly personalized and often secondary while others are constant across tumor types or within a tumor type; these last targets play, in most likelihood, a primary role in the disease and it is essential to diagnose them (even if only for their prognostic value). In addition, nothing prohibits the combination of specific drugs with cytotoxic or hormonal treatments (Nikanjam et al., 2016). The idea is to improve as much as possible the rational drug use to maximize the patient benefit. Many patients are dying from the toxic collateral effect of the chemotherapy; it would be a great success if the use of specific drugs in a standard therapy protocol could enable to decrease the dose of cytotoxic drugs and improve the therapy acceptance by patients in some specific cases in the context of theranostics. For this kind of exercise, an automated pipeline is needed and a clinical trial testing the validity of hubs as potential molecular targets is urgent.

The replication number that can be done for RNA-seq is another limitation given the still high cost of this technology. Thus, analyses as the one described in our manuscript are expected to be done only once per time in a time series for each patient. According to Barabasi’s theory (Barabási, 2016), hubs with the same connection rate are expected to have the same disarticulation effect on the signaling network. On a clinical basis, *p*-values (here critical value) may be adapted to the specific case of each patient. On the same line of reasoning, our methodology can be easily adapted taking into account more powerful bioinformatics tools and statistical analysis, but this issue is beyond the scope of this report. For such a methodology improvement, we believe that entropy is a good measure because it is universal, robust, and not dependent on sample size. Different combinations of normalization and statistical analyses as those reported by Li et al. (2020) can be compared in the same framework we presented here and in Conforte et al. (2019), by looking at how they may maximize the correlation coefficient of the negative relationship between entropy and OS. Of course, this depends on accepting the hypothesis that more aggressive tumors have more complex signaling networks, but again, this statement has been repeatedly claimed by several authors worldwide and along several years (Teschendorff and Severini, 2010; van Wieringen and van der Vaart, 2011; Breitkreutz et al., 2012; West et al., 2012; Banerji et al., 2015). If this hypothesis is true, the negative correlation between entropy and OS may serve as a calibration to study the optimization of RNA-seq methodologies and the influence of other factors in cancer development and dynamics.

Cancer is a genomic disease that affects DNA replication checkpoints through mutations of key oncogenes and suppressor genes (Lee and Muller, 2010). There are ten main hallmarks for cancer from which uncontrolled division is the key one (Hanahan and Weinberg, 2011). When the disease is taken at a late stage, it may have spread in the body through metastasis and secondary tumors may have different molecular profiles. In such late tumor stages, an approach of cancer therapy only based on personalized oncology would in most likelihood be unsuccessful (Ashdown et al., 2015). However, specific drugs could increase the patient benefit by supplementing traditional therapies based on cytotoxic drugs. As a consequence, the maximum benefit of a personalized oncology approach of solid tumor therapy based on a molecular phenotype diagnosis is in the early stages of malign cell multiplication. Despite its limitations, the phenotype approach of molecular diagnosis proposed here is needed for rational drug (or biopharmaceutical) therapy to maximize patient benefit.

At the moment, the methodology and the web site that we described here can be assimilated to *laboratory developed tests* (LDT). It is notorious that LDT for being a type of *in vitro* diagnostic test designed, manufactured, and used within a single laboratory is poorly supported by oncologists (8%) and pathologists (12%) because of the legitimate fair of innovation. Biomarkers and CDs strongly depend on the regulation by official organizations for their acceptance by health decision-makers (Novartis, 2020). However, barriers by regulation are no reason to stop the innovation necessary for progress. Otherwise, regulation fails with its purpose of protecting lives (see Carels et al., 2020 for a review).

### Web Application

System biology has gained considerable attention in medical sciences in the last decade thanks to the ever-increasing computer power. However, system biology models can be tricky to use or to interpret by non-experts in modeling. A recurrent question is how to integrate models into the physician daily lives such that they could best participate in their decision-making process. One potential solution, which seems to be the predominant one on the current state of the art, is by packing algorithms into software bundles and to make them available by user-friendly interfaces, such that little, or even no, expertise is required to use them. This is the paradigm we followed in this report.

The power and diversity of Angular programmed with TypeScript enable to expand the functionalities of the prototype proposed here in future versions, including the implementation of heavy calculations on the frontend side.

We chose MongoDB for storing genetic and medical records even if Galaxy has its own database system (postgreSQL). Our choice of MongoDB was motivated by the care of keeping coherence with MEAN stack, and also because of the power of MongoDB for Big Data storage. In addition, MongoDB is a non-relational database (NoSQL), which allows the storage of data in different formats within the same database.

Our implementation of online forms offers the possibility of creating new functions such as data validation. Data can be validated by comparing frontend to backend information through the database and making sure, for instance, that an entered e-mail does not already belong to someone else already registered in the system.

Finally, one common concern on web-programming is to minimize client communication with the server to maximize performance. For such purpose, we implemented a process of form validation on the frontend side. Since we are using FormBuilder (see for more details Fain and Moiseev, 2018), there are a set of built-in validation routines, and the possibility to easily create customized validation, thus any specific demand concerning data validation can be handled on future versions using the current source code.

## Conclusion

In a successive set of publications, we developed a rational methodology for the diagnosis of connection hubs among up-regulated genes of malignant subnetworks. This strategy is an application of graph theory, whose relevance has been mathematically proven by Albert et al. (2000). The inference of this theory into biological systems performed by Carels et al. (2015a) has been successfully validated on malignant cells by Tilli et al. (2016) and extended to tumor tissues by Conforte et al. (2019).

Here, in a translational oncology effort, we outlined a workflow that automated that research and allows its application to a large set of RNA-seq data to interact with public entities of the oncological sector, such as pharmaceutical companies, hospitals, diagnostic laboratories, public health care systems, and insurance groups around the world.

We belief that innovation in new translational solutions, like the one outlined here, is an imperative attribute of research centers; however, other agents such as (i) pharmaceutical companies may certainly help these initiatives with their experience concerning regulation, market barriers, financial support and (ii) startups whose processing speed and innovation potential were already well-documented (Blank and Dorf, 2012).

Herein, we aimed at transcending basic cancer inferences to bring a solution for clinical applications on a global scale.

## Data Availability Statement

Publicly available datasets were analyzed in this study. This data can be found here: https://galaxy.cdts.fiocruz.br/.

## Author Contributions

JP contributed for the development of the web application and wrote the corresponding sections. GS built the galaxy environment. TW wrote the Phython script. AC did the R analysis and contribute to the pipeline logic. DP prepared the medical forms. FS contributed with manuscript writing. NC wrote the Perl scripts, set the pipeline logic up, and wrote and managed the manuscript writing. All the authors contributed to the article and approved the submitted version.

## Conflict of Interest

The intellectual property of this research is protected by the Brazilian patent number BR1020150308191. The authors declare that the research was conducted in the absence of any commercial or financial relationships that could be construed as a potential conflict of interest.

## Footnotes

1 https://ourworldindata.org/cancer

2 https://pubmed.ncbi.nlm.nih.gov/?term=breast+cancer

3 https://www.nanostring.com

4 http://www.genome.jp/kegg

5 https://portal.gdc.cancer.gov

6 https://en.wikipedia.org/wiki/Lazy_loading. Accessed on 14/10/2020.

7 https://cancergenome.nih.gov/

8 https://portal.gdc.cancer.gov/

9 https://docs.gdc.cancer.gov/Data/Bioinformatics_Pipelines/Expression_mRNA_Pipeline/

10 https://www.ebi.ac.uk/ena/data/view/PRJEB2449

11 https://www.ebi.ac.uk/ena/data/view/PRJNA320473

12 https://www.ebi.ac.uk/arrayexpress/

13 ftp://ftp.ebi.ac.uk/pub/databases/intact/current/psimitab/intact-micluster.txt

14 https://gdc.cancer.gov/about-data/data-harmonization-and-generation/gdc-reference-files

15 https://www.ncbi.nlm.nih.gov/projects/CCDS/CcdsBrowse.cgi

16 https://mongoosejs.com/docs/

17 https://docs.mongodb.com/manual/sharding/

18 https://www.heroku.com/

19 http://teranostico.herokuapp.com/

20 https://www.npmjs.com/package/repository

21 https://material.angular.io/. Accessed on 14/10/2020.

22 http://www.passportjs.org/. Accessed on 14/10/2020.

23 https://docs.mongodb.com/manual/sharding/. Accessed on 14/10/2020.

24 https://www.mongodb.com/cloud/atlas. Accessed on 14/10/2020.

25 https://mongoosejs.com/docs/. Accessed on 14/10/2020

26 https://docs.mongodb.com/manual/sharding/. Accessed on 14/10/2020.

27 http://www.passportjs.org/. Accessed on 16/10/20.

28 https://www.npmjs.com/package/express-jwt. Accessed on 16/10/20.

29 Components in the Angular realm is a set of three files: CSS (appearance-related), TS (typescript, coding), and HTML (classical static page design file). A page is built from at least one component, which can independently interact with each one of the others (see Fain and Moiseev, 2018 for a more detailed discussion).

30 https://angular.io/guide/reactive-forms

31 Application Programming Interface. These routines are designed to access the database following some pre-defined rules such as token authentication.

32 https://www.npmjs.com/package/crypto-js

33 https://www.npmjs.com/package/dotenv

34 https://www.npmjs.com/package/bcrypt

## Supplementary Material

Supplementary Figure 1 Adaptative display according to device screen size (source: Fain and Moiseev, 2018).

Supplementary Figure 2 Flowchart of main form filling for exam request.

Supplementary Figure 3 Flowchart of outcome form filling.

Supplementary Figure 4 Authentication process. A *Guard* function double-check a user’s requisition and if access conditions are met the user is allowed to see the content of a requested page (green arrows). By contrast, if something went wrong (e.g., token expired), the access is denied (red arrow).

Supplementary Figure 5 Login card.

Supplementary Figure 6 Example of main form options.

Supplementary Figure 7 Example of outcome form being implemented.

Supplementary Figure 8 The page #1 of the main form given as an example.

Supplementary Figure 9 Receipt of main form submission.

Supplementary Figure 10 Form after retrieval from the Dashboard (the entire form does not fit the page).

Supplementary Table 1 Scaling of pipeline from Figure 2B (entropy) and Figure 6B (PTTCS) using GDC read counts (see Figure 7).
