# Supplementary figures and images for: Galaxy and MEAN Stack to Create a User-Friendly Workflow for the Rational Optimization of Cancer Chemotherapy
Source: Front Genet. 2021 Feb 18;12:624259. doi: 10.3389/fgene.2021.624259 (PMC7935533; doi:10.3389/fgene.2021.624259)

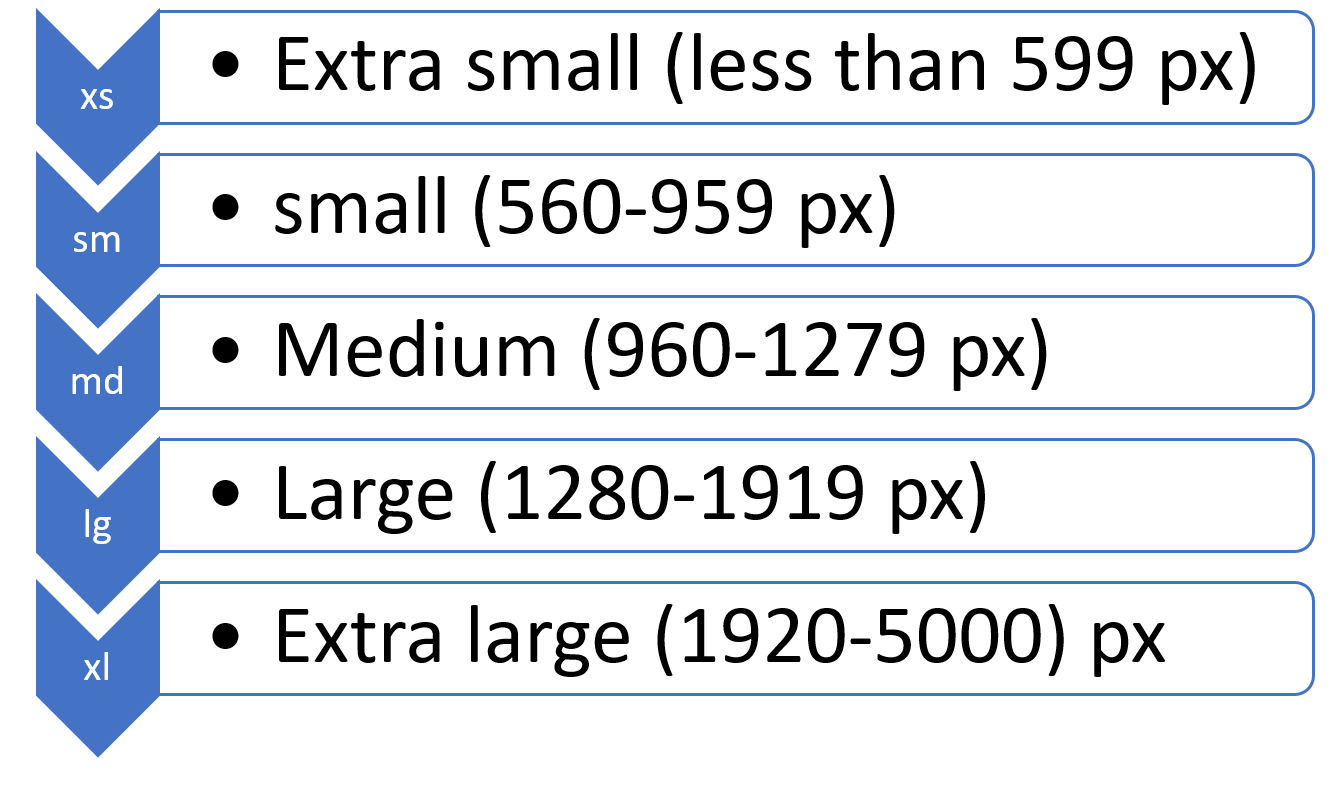

Supplement: Supplementary Figure 1 — Adaptative display according to device screen size (source: Fain and Moiseev, 2018). [file Image_1.TIF]

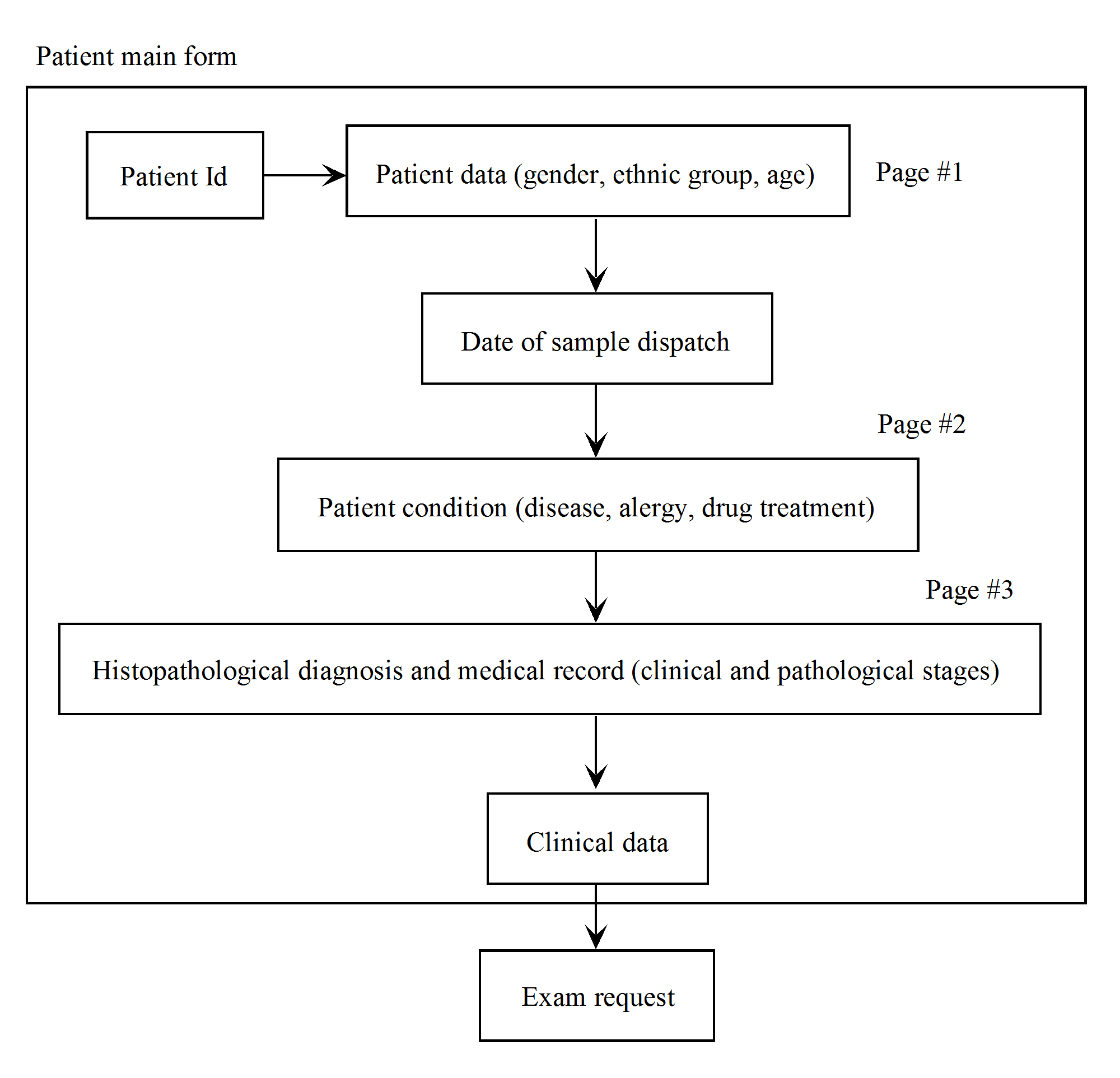

Supplement: Supplementary Figure 2 — Flowchart of main form filling for exam request. [file Image_2.TIF]

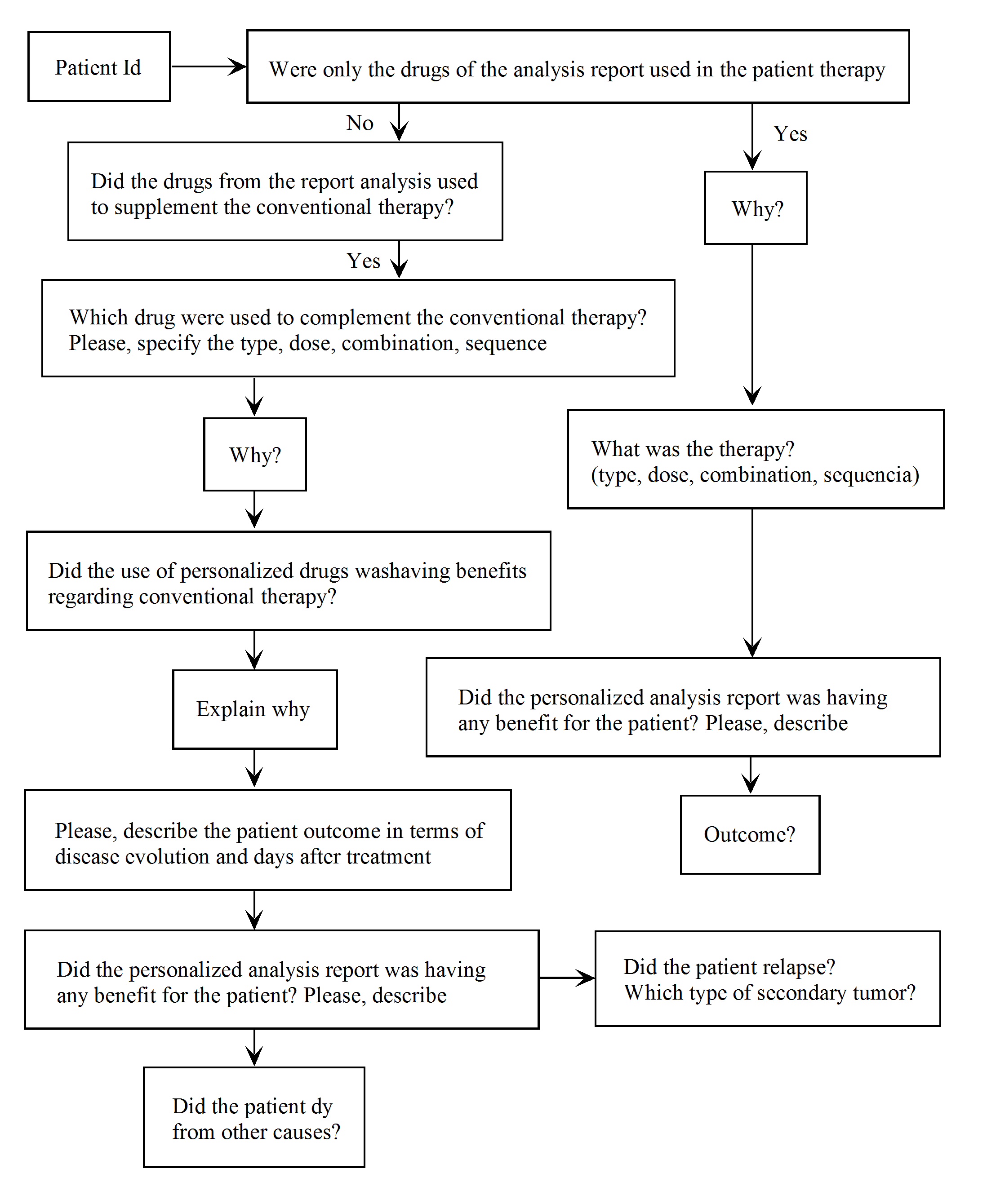

Supplement: Supplementary Figure 3 — Flowchart of outcome form filling. [file Image_3.TIF]

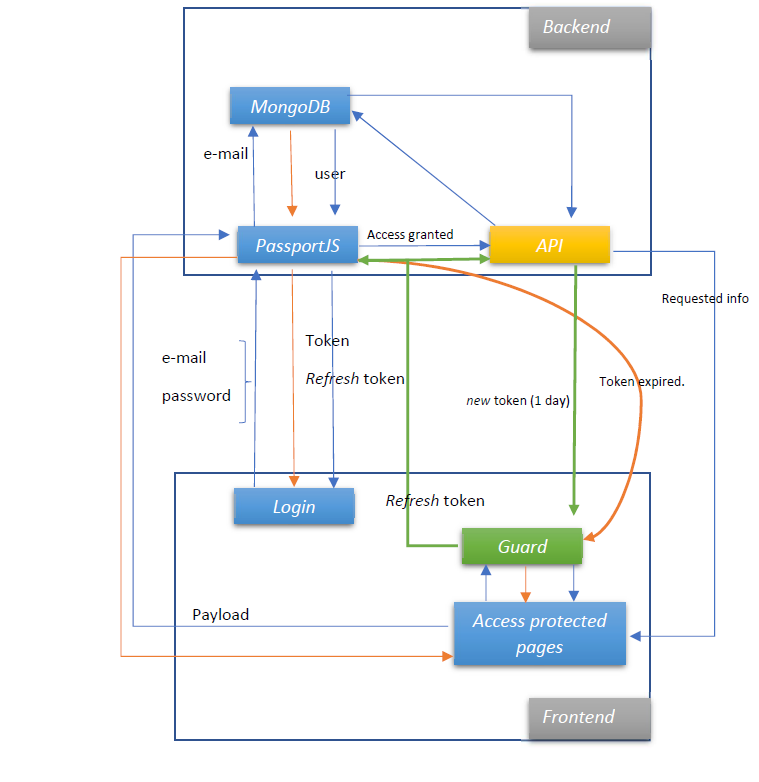

Supplement: Supplementary Figure 4 — Authentication process. A Guard function double-check a user’s requisition and if access conditions are met the user is allowed to see the content of a requested page (green arrows). By contrast, if something went wrong (e.g., token expired), the access is denied (red arrow). [file Image_4.TIF]

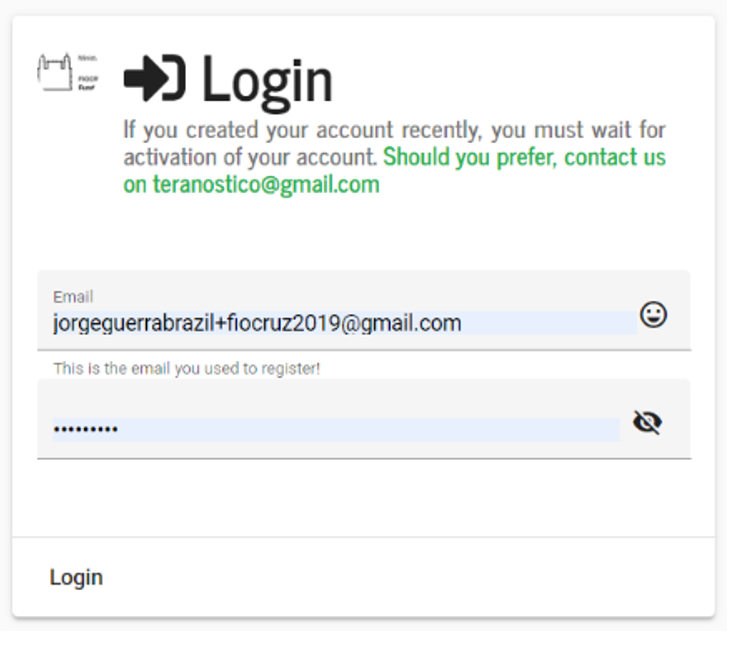

Supplement: Supplementary Figure 5 — Login card. [file Image_5.TIF]

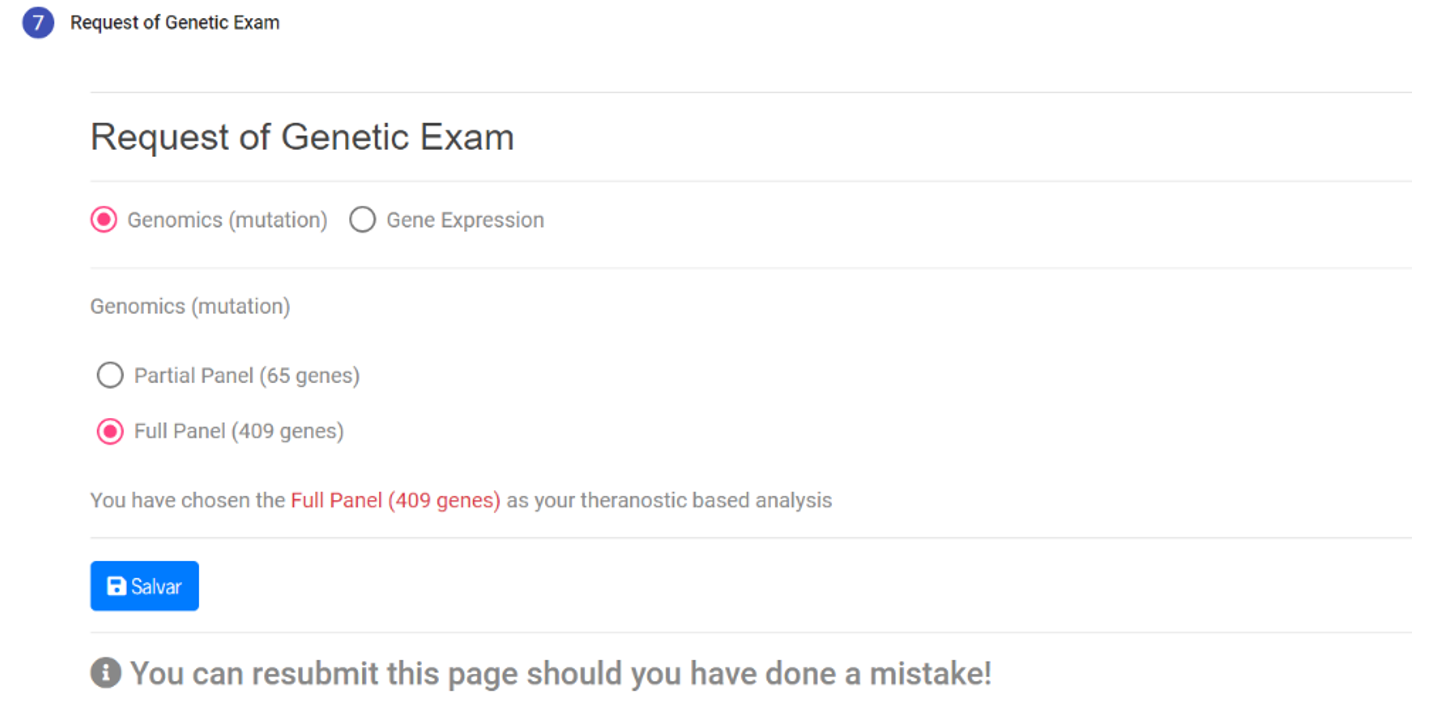

Supplement: Supplementary Figure 6 — Example of main form options. [file Image_6.TIF]

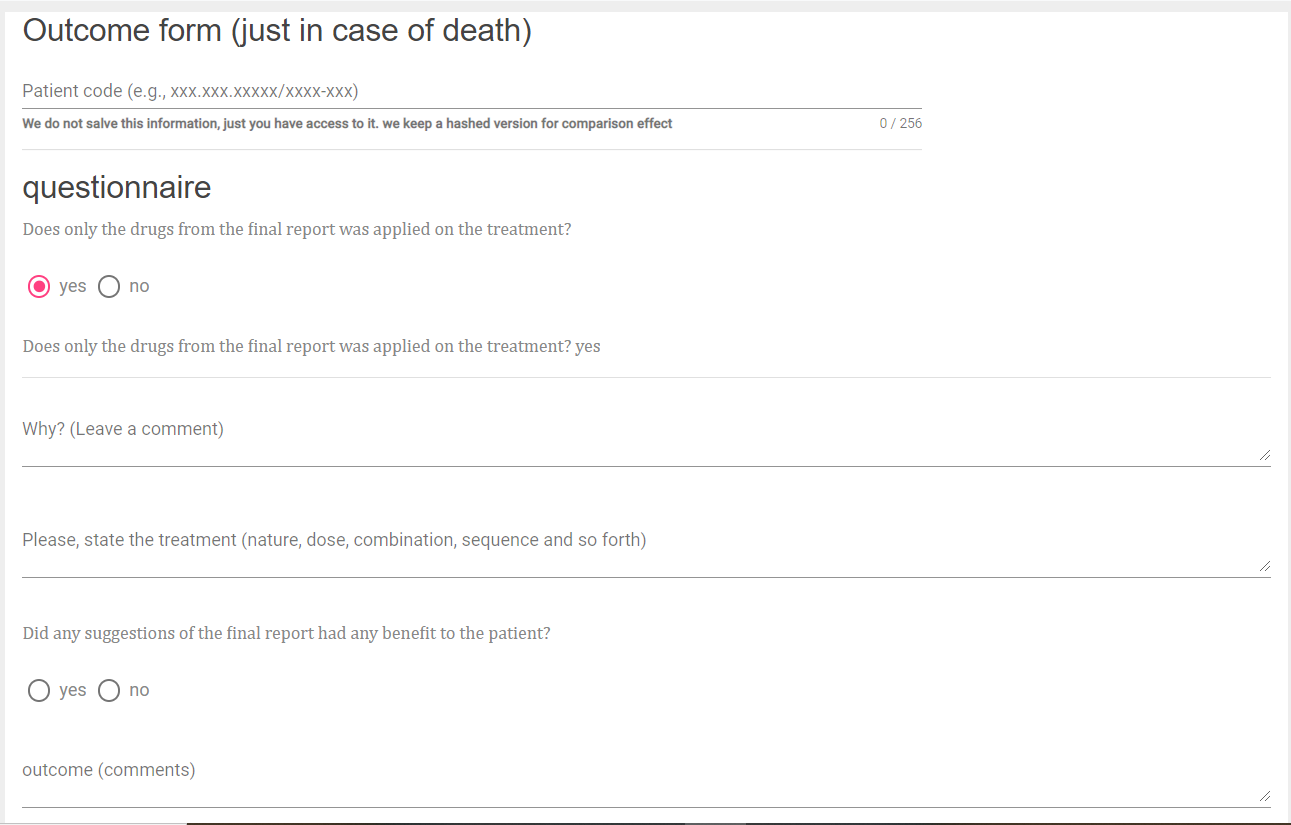

Supplement: Supplementary Figure 7 — Example of outcome form being implemented. [file Image_7.TIF]

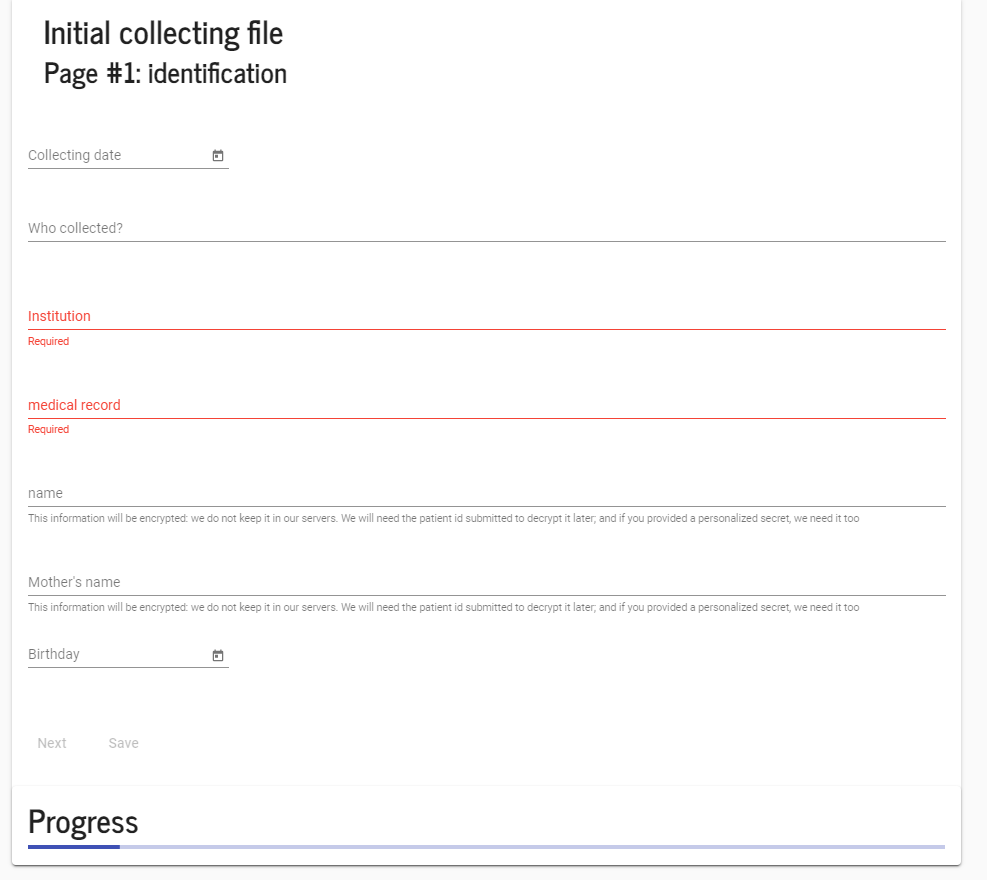

Supplement: Supplementary Figure 8 — The page #1 of the main form given as an example. [file Image_8.TIF]

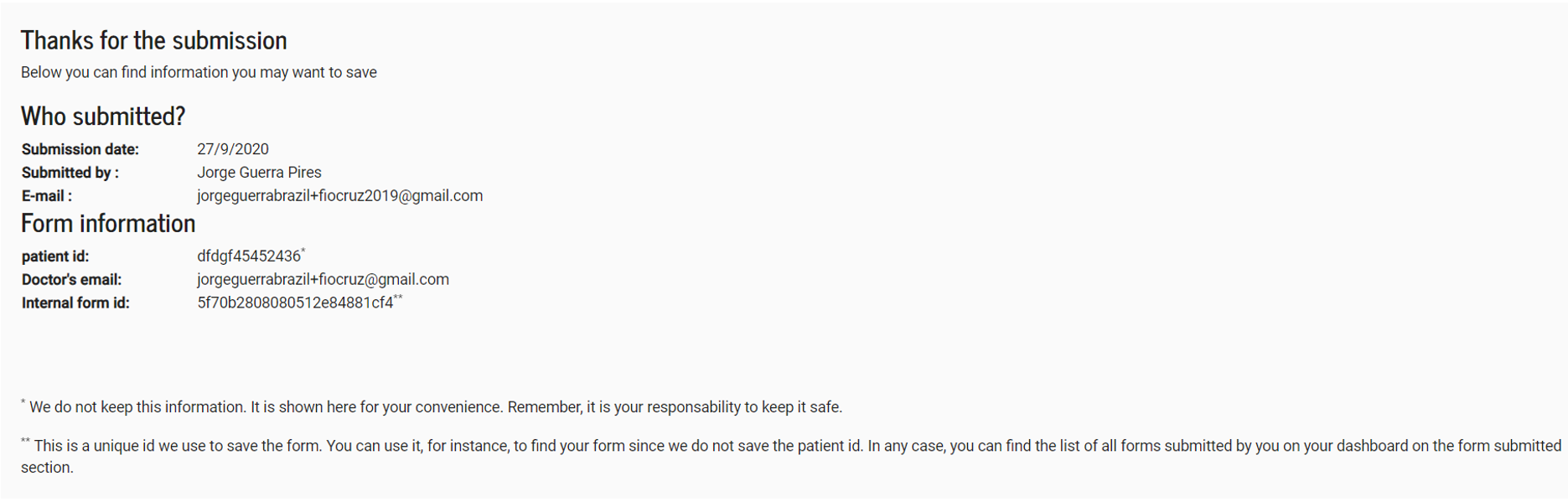

Supplement: Supplementary Figure 9 — Receipt of main form submission. [file Image_9.TIF]

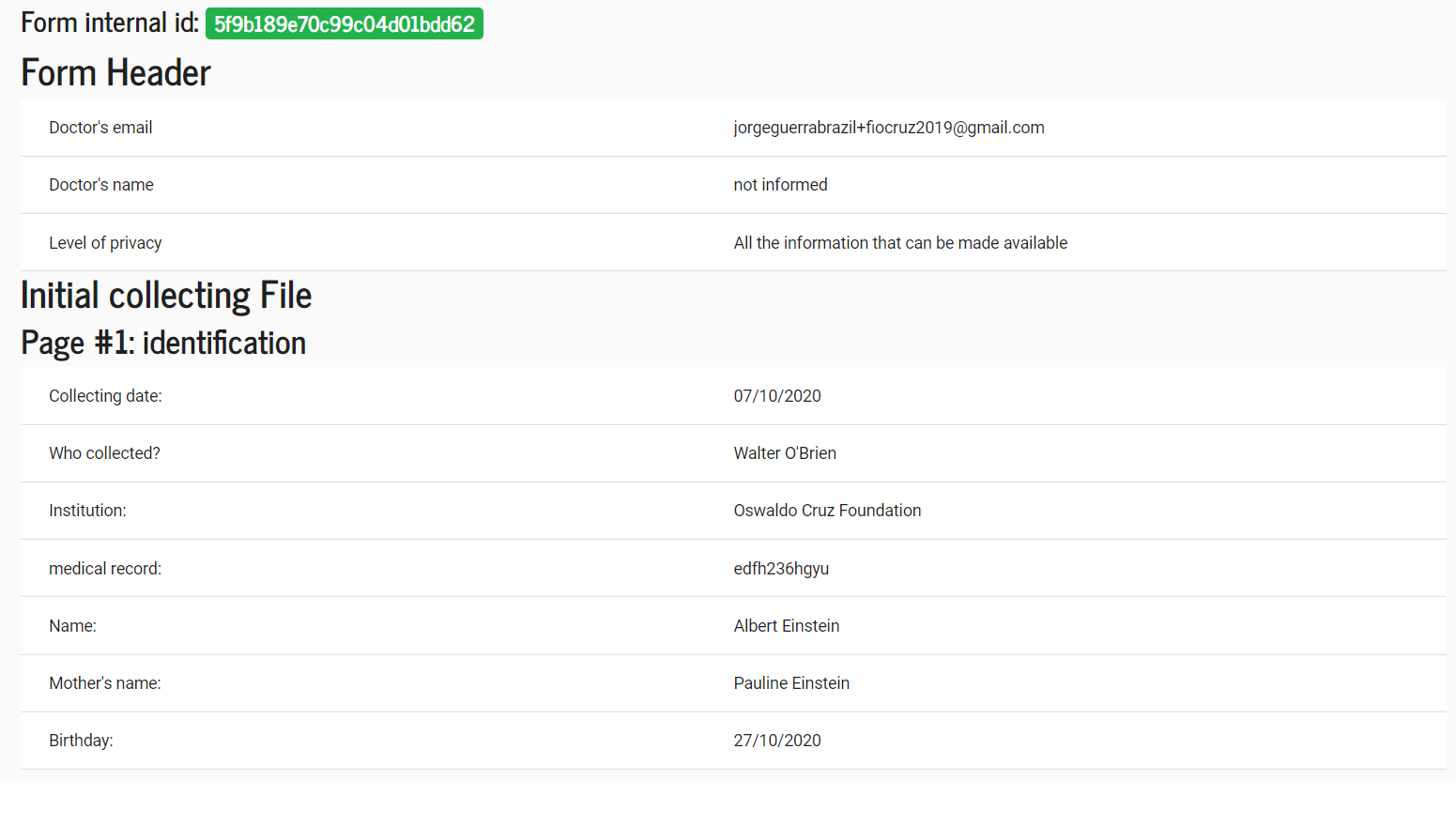

Supplement: Supplementary Figure 10 — Form after retrieval from the Dashboard (the entire form does not fit the page). [file Image_10.TIF]
