# Supplementary material for: Galaxy and MEAN Stack to Create a User-Friendly Workflow for the Rational Optimization of Cancer Chemotherapy
Source: Front Genet. 2021 Feb 18;12:624259. doi: 10.3389/fgene.2021.624259 (PMC7935533; doi:10.3389/fgene.2021.624259)
Supplement: Supplementary Table 1 — Scaling of pipeline from Figure 2B (entropy) and Figure 6B (PTTCS) using GDC read counts (see Figure 7). [file Table_1.DOCX]

**Supplementary Table 1**. Scaling of pipeline from Figs. 2B (entropy) and 6B (PTTCS) using GDC read counts (see Fig. 7).

| Pipeline type | Stat. | Cancer type | Patient number | | |  |  |  |  |  |  |  | Correl. | Linear fitting |
| --- | --- | --- | --- | --- | --- | --- | --- | --- | --- | --- | --- | --- | --- | --- |
|  |  |  | 5 | 10 | 15 | 20 | 25 | 30 | 35 | 40 | 45 |  |  |  |
| Entropy |  | LUSC | 154^†^ | 303 | 459 | 595 | 735 | 867 | 1013 | 1141 | 1291 |  | 1.000 | y=28.1x+24.9 |
|  |  | STAD |  |  | 477 |  | 779 |  |  |  |  |  |  | y=30.2x+24.0 |
|  |  | LIHC |  |  | 460 |  | 730 |  |  |  |  |  |  | y=27.0x+55.0 |
|  | Av. |  |  |  |  |  | 748 |  |  |  |  |  |  |  |
|  | SEM |  |  |  |  |  | 16 |  |  |  |  |  |  |  |
|  |  |  |  |  |  |  |  |  |  |  |  |  |  |  |
|  |  | PRAD | 170 | 248 | 356 | 510 | 611 | 734 | 836 | 983 | 1074 |  | 0.999 | y=23.4x+28.3 |
|  |  | THCA |  |  | 343 |  | 565 |  |  |  |  |  |  | y=22.2x+10.0 |
|  |  | KIRC |  |  | 392 |  | 606 |  |  |  |  |  |  | y=21.4x+71.0 |
|  | Av. |  |  |  |  |  | 594 |  |  |  |  |  |  |  |
|  | SEM |  |  |  |  |  | 15 |  |  |  |  |  |  |  |
|  | t-test |  |  |  |  |  | 5** |  |  |  |  |  |  |  |
|  |  |  |  |  |  |  |  |  |  |  |  |  |  |  |
| PTTCS |  | LUSC | 97 | 184 | 291 | 376 | 472 | 573 | 648 | 756 | 834 |  | 1.000 | y=18.6x+5.2 |
|  |  | STAD |  |  | 304 |  | 482 |  |  |  |  |  |  | y=17.8x+37.0 |
|  |  | LIHC |  |  | 300 |  | 464 |  |  |  |  |  |  | y=16.4x+53.4 |
|  | Av. |  |  |  |  |  | 473 |  |  |  |  |  |  |  |
|  | SEM |  |  |  |  |  | 5 |  |  |  |  |  |  |  |
|  |  |  |  |  |  |  |  |  |  |  |  |  |  |  |
|  |  | PRAD | 97 | 168 | 268 | 368 | 433 | 518 | 586 | 684 | 767 |  | 0.999 | y=16.7x+13.5 |
|  |  | THCA |  |  | 263 |  | 420 |  |  |  |  |  |  | y=15.7x+27.5 |
|  |  | KIRC |  |  | 278 |  | 428 |  |  |  |  |  |  | y=15.0x+53.0 |
|  | Av. |  |  |  |  |  | 427 |  |  |  |  |  |  |  |
|  | SEM |  |  |  |  |  | 4 |  |  |  |  |  |  |  |
|  | t-test |  |  |  |  |  | 5** |  |  |  |  |  |  |  |

**significant at α≤0.01 for *k* = 4 degrees of freedom. ^†^Time in seconds. Av.: Average. SEM: Standard error of the mean. Stat.: Statistics. Correl.: Correlation.
